# Fluctuations of micronutrients during pregnancy in Lianyungang, Jiangsu, China: A prospective longitudinal cohort study

**DOI:** 10.1371/journal.pone.0360813

**Published:** 2026-10-16

**Authors:** Lianshuai Zheng, Xiaolun Xu, Xueyuan Rong, Jian Luo, Guoqing Chen, Cheng Zhao

**Affiliations:** 1 Lianyungang Maternal and Child Health Hospital, Lianyungang, Jiangsu, China; 2 Lianyungang Research Institute for Women’s and Children’s Health, Lianyungang Maternal and Child Health Hospital, Lianyungang, Jiangsu, China; 3 Kangda College of Nanjing Medical University, Lianyungang, Jiangsu, China; King Faisal Specialist Hospital and Research Center, SAUDI ARABIA

## Abstract

Pregnancy is accompanied by substantial physiological adaptations that may alter maternal micronutrient concentrations. A prospective longitudinal pregnancy-neonatal cohort of 51 healthy pregnant women was established at 6–8 weeks of gestation in Lianyungang, Jiangsu, China, confirmed by fetal heart and bud on B-ultrasound. Pregnancy complications, outcomes, and neonatal health at day 42 were confirmed. Forty-three women who completed all assessments and had uncomplicated pregnancies with favorable maternal and neonatal outcomes were included in the final analysis. Whole-blood trace elements and serum vitamins were quantified during the first trimester (12.3 ± 0.9 weeks), second trimester (25.6 ± 1.2 weeks), and third trimester (32.7 ± 2.3 weeks) using ICP-MS and UHPLC-MS/MS. The results showed that copper, calcium, and vitamin D peaked in the second trimester, whereas iron, zinc, magnesium, and lead reached their nadir in the second trimester. Meanwhile, manganese and vitamins E and K increased progressively throughout gestation. In contrast, several B vitamins showed an overall downward trend, while vitamin B12 remained relatively stable. These findings demonstrate clear trimester-specific changes in maternal micronutrient levels during healthy pregnancy. Given the relatively small sample size, these longitudinal data provide preliminary evidence to inform the establishment of region-specific and trimester-specific reference intervals and deficiency thresholds for micronutrients in healthy pregnant women.

## 1. Introduction

Pregnancy is a unique physiological state characterized by plasma volume expansion, enhanced maternal-fetal nutrient transfer, altered renal excretion, and increased metabolic demand. Nevertheless, reference ranges for many nutritional and metabolic biomarkers are still based on non-pregnant populations. Among healthy pregnant women, many circulating biomarkers change substantially with advancing gestation. Therefore, direct application of reference ranges established in non-pregnant adults, or reliance on a single reference range throughout pregnancy, may result in inaccurate assessment of maternal nutritional status. Previous studies have shown that serum ferritin and hemoglobin levels decrease markedly in the second trimester [1,2], whereas D-dimer levels rise progressively throughout pregnancy [3]. Overall, these observations indicate that biomarkers measured during pregnancy should be interpreted in the context of gestational physiology. Ideally, separate reference intervals and deficiency cutoffs should be established for the first, second, and third trimesters.

Although the assessment of micronutrient status currently relies mainly on peripheral blood samples, trimester-specific reference ranges for the first, second, and third trimesters have not yet been established. In addition, micronutrient levels are influenced by multiple factors, including dietary patterns [4], living environment [5], ethnic background [6], and socioeconomic status [7]. Although studies on micronutrients and vitamins during pregnancy are increasing, most of the existing literature is based on cross-sectional designs, which cannot adequately capture their dynamic changes throughout gestation.

This study adopted a longitudinal cohort design covering the first, second, and third trimesters, thereby providing a more systematic and comprehensive framework for understanding these dynamic alterations during pregnancy. This study aimed to systematically evaluate the changing patterns of essential trace elements (Cu, Fe, Zn, Ca, Mg, and Mn), Pb as well as fat-soluble and water-soluble vitamins throughout pregnancy. By characterizing the distribution ranges of these biomarkers across the three trimesters in healthy pregnant women from this region, our findings provide preliminary data for establishing trimester-specific reference ranges and deficiency cutoffs, highlighting their potential clinical significance.

## 2. Methods

### 2.1. Participants

This prospective pregnancy-neonatal cohort study was conducted at Lianyungang Maternal and Child Health Hospital in Lianyungang, Jiangsu, China. From May 2020 to June 2021, 130 pregnant women were initially screened for eligibility at 6−8 weeks of gestation. Of these, 79 were excluded because of diabetes (n = 20), thyroid disorders (n = 16), threatened miscarriage (n = 15), smoking or secondhand smoke exposure (n = 10), special dietary patterns (n = 7), a history of adverse pregnancy outcomes (n = 6), or anemia (n = 5). The remaining 51 eligible healthy pregnant women were enrolled and prospectively followed throughout pregnancy. Enrollment during early pregnancy was confirmed by the presence of fetal cardiac activity and an embryonic bud on B-mode ultrasonography. All participants received routine antenatal care. After enrollment, participants were advised to take folic acid (0.4–0.8 mg/day) and calcium (600 mg/day). Adherence to supplementation was assessed by self-report during follow-up, and all 43 participants included in the final analysis reported taking both supplements as recommended. Information on self-initiated use of vitamin D or other nutritional supplements was not systematically collected. Pregnancy complications, pregnancy outcomes, and neonatal health at 42 days postpartum were recorded. The maternal and neonatal characteristics of the study participants are summarized in Table 1.

**Table 1:** Maternal and neonatal characteristics.

Characteristic	Mean ± SD or n (%)
Maternal age (years)	27.9 ± 3.5
Maternal height (cm)	162.0 ± 5.5
Pre-pregnancy weight (kg)	60.2 ± 9.6
Pre-pregnancy BMI (kg/m²)	22.9 ± 3.2
Maternal weight at delivery (kg)	76.4 ± 11.5
Gestational weight gain (kg)	16.2 ± 4.1
Education level, n (%)	
High school/vocational school or below	12 (27.9)
Junior college	16 (37.2)
Bachelor’s degree or higher	15 (34.9)
Occupation, n (%)	
Unemployed	12 (27.9)
Company employee	11 (25.6)
Public sector/professional	10 (23.3)
Other	10 (23.3)
Annual income (RMB), n (%)	
<50000	19 (44.2)
50000-100000	15 (34.9)
> 100000	9 (20.9)
Gravidity (n)	1.9 ± 1.3
Parity (n)	0.4 ± 0.6
Blood collection time (weeks)	
First trimester	12.3 ± 0.9
Second trimester	25.6 ± 1.2
Third trimester	32.7 ± 2.3
Gestational age at delivery (weeks)	39.7 ± 0.9
Infant sex, n (%)	
Male	22(51.2)
Female	21(48.8)
Neonatal birth weight (g)	3545 ± 386
Apgar score (10 min)	10

Whole-blood concentrations of Cu, Fe, Zn, Ca, Mg, Mn, and Pb, together with serum concentrations of vitamins A, B1, B2, B9, B12, D, D2, D3, E, and K, were measured in the first, second, and third trimesters. Total vitamin D was calculated as the sum of vitamin D2 and vitamin D3.

The inclusion criteria were as follows: absence of underlying medical conditions; no known vitamin or trace element deficiencies; adherence to a normal diet according to the China Maternal and Infant Dietary Guidelines (2022); no special dietary practices; no significant occupational or environmental exposure to pollutants; no pregnancy complications during the index pregnancy; no adverse pregnancy outcomes, including miscarriage, preterm birth, stillbirth, or intrauterine fetal death, as confirmed at the 42-day postpartum follow-up; and normal neonatal findings at the 42-day postnatal examination.

The exclusion criteria were as follows: multiple gestation; immune, infectious, or endocrine disorders; a history of genetic disorders or adverse pregnancy outcomes attributable to genetic factors; smoking, alcohol abuse, or substance misuse; exposure to toxic chemicals; use of medications known to affect micronutrient metabolism; non-adherence to the China Maternal and Infant Dietary Guidelines (2022); pregnancy complications or adverse pregnancy outcomes during the current pregnancy; and neonatal disease or abnormal findings identified at birth or at the 42-day postnatal examination. The flow of participant selection process is presented in Fig 1.

**Fig 1:**
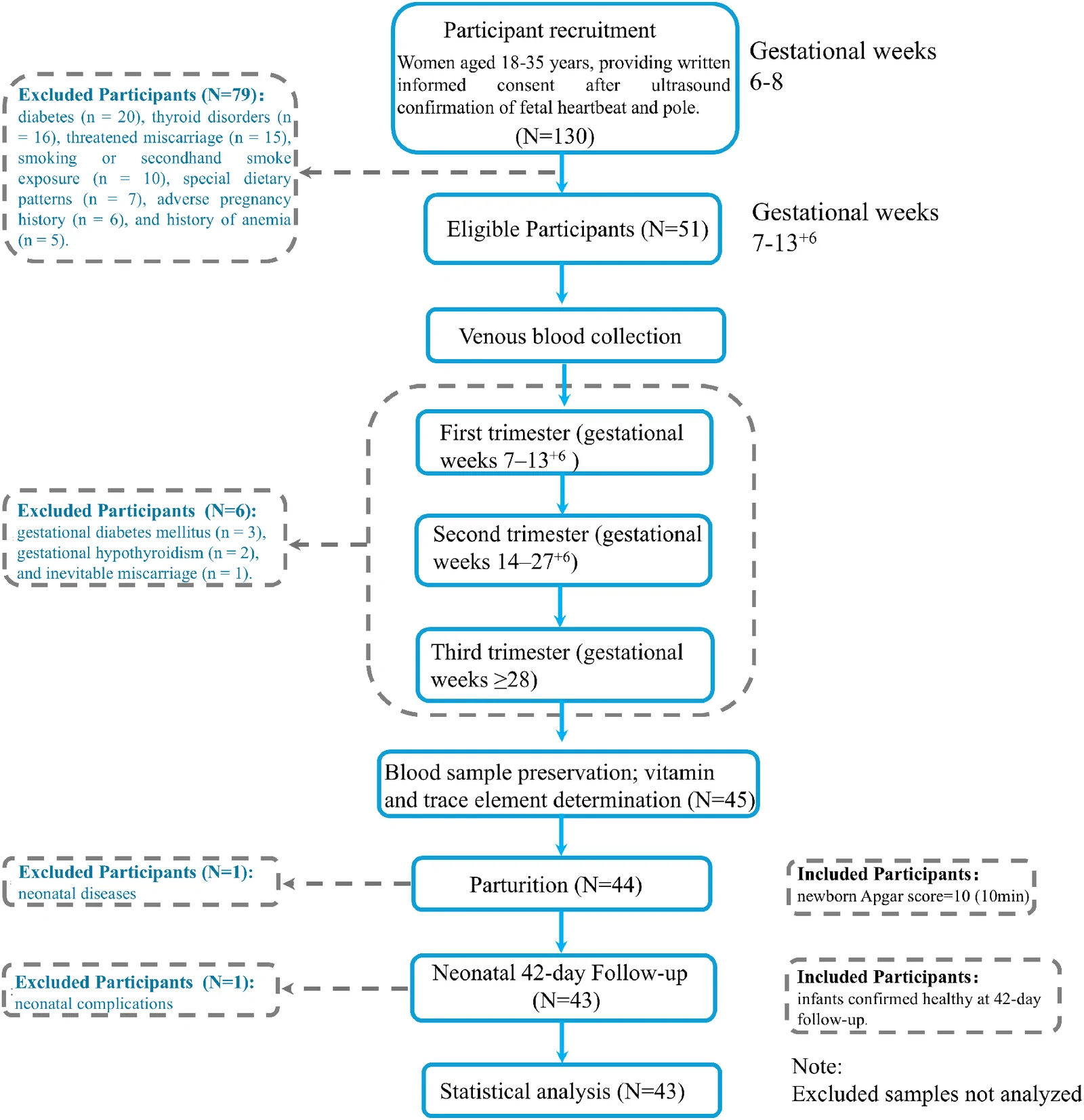
Flowchart of this study.

Among the 51 enrolled women, six were excluded during pregnancy because of gestational diabetes mellitus (n = 3), gestational hypothyroidism (n = 2), or inevitable miscarriage (n = 1). Of the 45 remaining participants, one was excluded after neonatal disease was diagnosed at delivery, and another after neonatal complications emerged during the 42-day follow-up. Thus, 43 participants met the final eligibility criteria and were included in the statistical analysis. This study was conducted in accordance with the Declaration of Helsinki. Written informed consent was obtained from all participants before enrollment. The study protocol was approved by the Ethics Committee of Lianyungang Maternal and Child Health Hospital (LYG-ME2019020).

### 2.2. Peripheral blood collection

Based on gestational age confirmed by ultrasonography at enrollment, peripheral blood samples were collected in the first trimester (12.3 ± 0.9 weeks), second trimester (25.6 ± 1.2 weeks), and third trimester (32.7 ± 2.3 weeks). The first trimester was defined as the period from the last menstrual period to the end of gestational week 13, the second trimester as gestational weeks 14–27, and the third trimester as gestational week 28 to delivery.

Participants fasted for more than 4 h before blood collection. For trace element analysis, 2 mL of peripheral blood was collected into an EDTA-K2 tube, gently mixed, aliquoted, and stored at −80 °C until analysis. For vitamin analysis, an additional 2 mL of peripheral blood was collected into a serum separation tube, centrifuged within 2 h, and the serum was aliquoted and stored at −80 °C until analysis.

### 2.3. Trace element and vitamin measurement

Whole-blood trace elements were measured by inductively coupled plasma mass spectrometry using an Agilent 7900 system (Agilent Technologies, USA). Briefly, 100 μL of whole blood was mixed with 1900 μL of diluent containing 0.1% nitric acid and 0.1% Triton X-100 and then analyzed. Concentrations of Cu, Fe, Zn, Ca, Mg, Mn, and Pb were quantified using the standard curve method.

Serum vitamins were measured by ultra-high-performance liquid chromatography-tandem mass spectrometry using an AB SCIEX Triple Quad 4500 or API 5500 system. Samples were prepared by protein precipitation with methanol or acetonitrile containing internal standards. For fat-soluble vitamins, the supernatants were further extracted with n-hexane before analysis. For water-soluble vitamins, the processed samples were freeze-dried before analysis.

Method validation and quality control were performed according to the laboratory’s standard operating procedures. Quality control materials were included in each analytical batch for both assays. Analytical runs were accepted only when calibration, recovery, and quality control results met predefined criteria. All analytical runs included in this study satisfied these requirements. Detailed quality-control data are presented in S1 Table.

### 2.4. Statistical analysis

All analyses were performed in R version 4.5.0. Concentrations of trace elements and vitamins were summarized as mean ± standard deviation for each trimester. Gestational week at sampling was also summarized as mean ± standard deviation within each trimester. Potential outliers were identified using Tukey’s 1.5 × IQR rule (values below Q1 - 1.5 × IQR or above Q3 + 1.5 × IQR). Only one manganese concentration (42.8 μg/L) was excluded and treated as missing in the manganese analysis, while other observations were retained.

Given the repeated-measures design, longitudinal changes were analyzed using linear mixed-effects models, with participant ID specified as a random intercept to account for within-subject correlation. To characterize gestational trajectories, gestational week was included as a centered continuous variable together with its quadratic term. A separate model including trimester as a categorical fixed effect was fitted to compare trimester-specific differences.

Overall trimester effects were assessed using F-tests, and pairwise comparisons were performed using estimated marginal means with Bonferroni adjustment. Both unadjusted and Bonferroni-adjusted P values are presented in S2 Table. Fixed-effect estimates and 95% confidence intervals were calculated using the Wald method. Analyses were performed using the lme4, lmerTest, emmeans, and ggplot2 packages. A two-sided *P* value <0.05 was considered statistically significant.

## 3. Results

### 3.1. Fluctuations in trace elements during pregnancy

Maternal copper levels were significantly higher in the second than in the first trimester, increasing by approximately 15.4%, and remained significantly elevated in the third trimester at approximately 15.9% above the first-trimester level. Iron levels decreased significantly by approximately 11.1% from the first to the second trimester and subsequently increased by approximately 7.8% from the second to the third trimester. Zinc levels increased significantly by approximately 11.9% from the second to the third trimester. Calcium levels increased significantly by approximately 3.0% from the first to the second trimester. Magnesium levels decreased significantly by approximately 10.4% from the first to the second trimester and increased significantly by approximately 4.1% from the second to the third trimester, although third-trimester levels remained significantly 6.8% lower than first-trimester levels. Manganese levels increased significantly throughout pregnancy, rising by approximately 15.1% from the first to the second trimester and by a further 26.1% from the second to the third trimester, corresponding to an overall increase of 45.2%. Detailed results for maternal whole-blood trace element concentrations during pregnancy are shown in Fig 2 and Table 2.

**Table 2:** Trimester-specific concentrations of trace elements and vitamins during pregnancy.

Analyte	First trimester		Second trimester		Third trimester	
	Mean (SD)	EMM [95% CI]	Mean (SD)	EMM [95% CI]	Mean (SD)	EMM [95% CI]
Copper (μg/L)	1278.96 (131.35)	1278.96 [1232.45, 1325.46]	1475.50 (140.98)	1475.50 [1428.99, 1522.01]	1482.63 (182.81)	1482.63 [1436.12, 1529.14]
Iron (mg/L)	430.53 (34.61)	430.53 [416.55, 444.51]	382.70 (47.69)	382.70 [368.71, 396.68]	412.68 (54.36)	412.68 [398.69, 426.66]
Zinc (mg/L)	5.62 (0.56)	5.62 [5.40, 5.85]	5.37 (0.76)	5.37 [5.15, 5.60]	6.01 (0.85)	6.01 [5.79, 6.23]
Calcium (mg/L)	60.80 (2.57)	60.80 [59.61, 61.99]	62.64 (4.55)	62.64 [61.45, 63.83]	60.97 (4.38)	60.97 [59.78, 62.16]
Magnesium (mg/L)	35.07 (2.72)	35.07 [34.26, 35.88]	31.41 (2.56)	31.41 [30.60, 32.23]	32.69 (2.75)	32.69 [31.87, 33.50]
Manganese (μg/L)^a^	13.73 (3.27)	13.95 [12.55, 15.34]	15.81 (4.13)	15.81 [14.42, 17.20]	19.94 (5.72)	19.94 [18.55, 21.33]
Lead (μg/L)	7.72 (1.91)	7.72 [7.01, 8.44]	7.08 (2.22)	7.08 [6.36, 7.79]	8.26 (2.85)	8.26 [7.54, 8.97]
Vitamin A (ng/mL)	399.46 (67.54)	399.46 [376.12, 422.79]	383.97 (88.82)	383.97 [360.64, 407.30]	347.26 (73.14)	347.26 [323.93, 370.60]
Vitamin B1 (ng/mL)	1.50 (0.52)	1.50 [1.34, 1.65]	1.60 (0.56)	1.60 [1.44, 1.75]	1.44 (0.43)	1.44 [1.28, 1.59]
Vitamin B2 (ng/mL)	10.88 (9.51)	10.88 [8.48, 13.27]	8.56 (7.45)	8.56 [6.17, 10.96]	7.71 (6.15)	7.71 [5.32, 10.11]
Vitamin B9 (ng/mL)	32.25 (9.16)	32.25 [29.04, 35.47]	22.34 (10.51)	22.34 [19.12, 25.56]	20.39 (11.91)	20.39 [17.17, 23.60]
Vitamin B12 (ng/mL)	0.22 (0.10)	0.22 [0.19, 0.25]	0.19 (0.09)	0.19 [0.16, 0.22]	0.19 (0.11)	0.19 [0.16, 0.22]
Vitamin D (ng/mL)	16.50 (7.81)	16.50 [14.13, 18.88]	21.97 (7.14)	21.97 [19.59, 24.34]	20.54 (8.50)	20.54 [18.17, 22.92]
Vitamin D2 (ng/mL)	0.52 (0.45)	0.52 [−0.26, 1.30]	2.00 (3.02)	2.00 [1.22, 2.78]	2.00 (3.26)	2.00 [1.22, 2.78]
Vitamin D3 (ng/mL)	15.99 (7.81)	15.99 [13.61, 18.36]	19.97 (7.08)	19.97 [17.59, 22.35]	18.55 (8.54)	18.55 [16.17, 20.92]
Vitamin E (μg/mL)	9.77 (1.96)	9.77 [9.00, 10.55]	13.45 (2.50)	13.45 [12.67, 14.23]	15.06 (3.09)	15.06 [14.29, 15.84]
Vitamin K (ng/mL)	0.52 (0.33)	0.52 [0.30, 0.75]	0.81 (0.62)	0.81 [0.58, 1.04]	1.35 (1.11)	1.35 [1.12, 1.57]

Values are presented as descriptive means (SDs) and estimated marginal means (EMMs) with 95% confidence intervals (CIs) derived from linear mixed-effects models. ^a^ One manganese value (42.8 μg/L) was excluded as an outlier.

**Fig 2:**
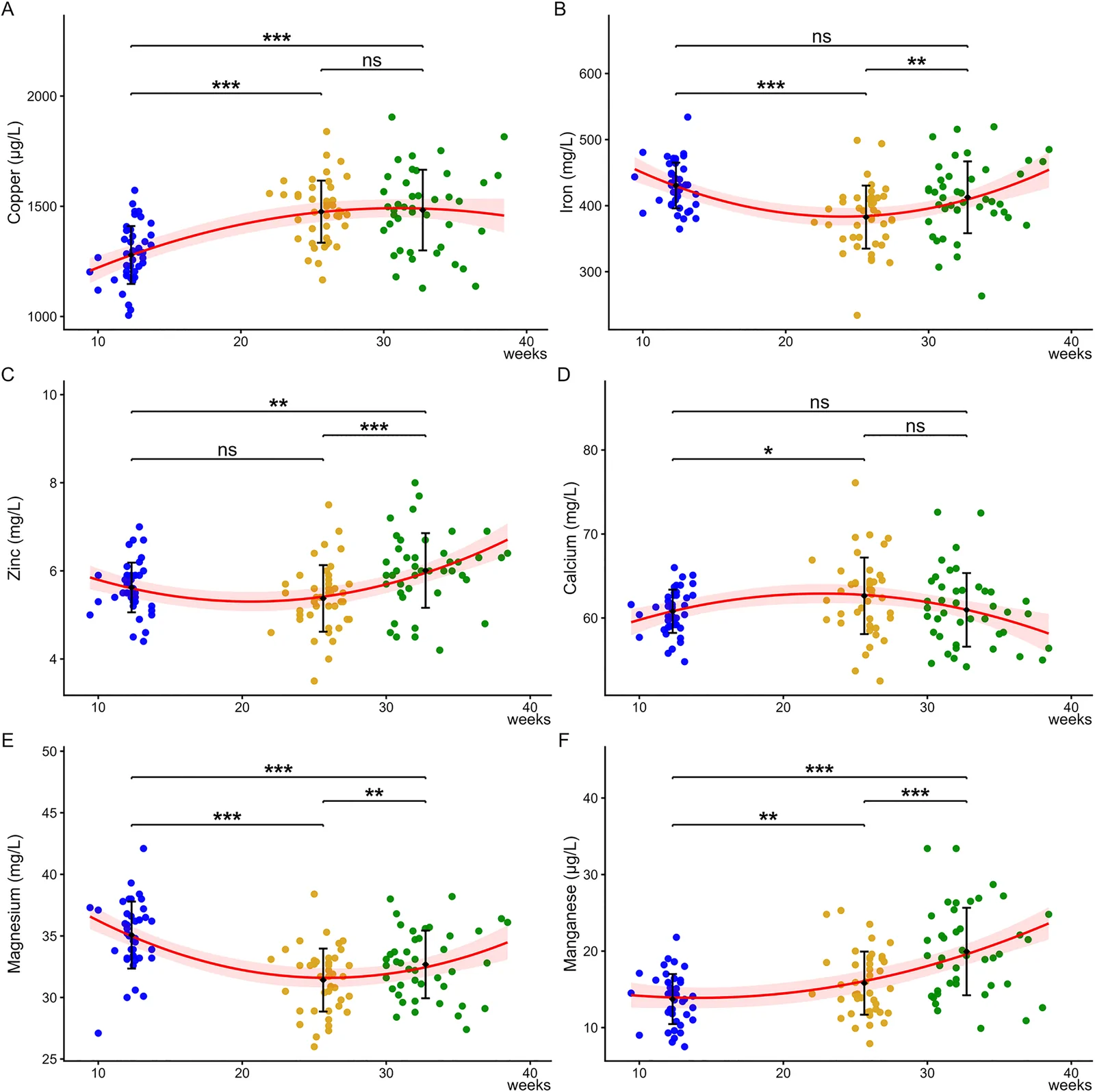
Trace elements in maternal whole blood. (A) Copper; (B) Iron; (C) Zinc; (D) Calcium; (E) Magnesium; (F) Manganese; (G) Lead. First trimester: blue; Second trimester: yellow; Third trimester: green. After Bonferroni correction, *P < 0.05, **P < 0.01, ***P < 0.001, and not significant (ns).

### 3.2. Fluctuations in fat-soluble vitamins during pregnancy

Maternal vitamin A levels decreased significantly by approximately 9.6% from the second to the third trimester, with the third-trimester levels approximately 13.1% lower than first-trimester levels. Total vitamin D increased significantly by approximately 33.2% from the first to the second trimester, with the third-trimester levels remaining 24.5% higher than the first-trimester levels. Vitamin D2 increased significantly by approximately 284.6% from the first to the second trimester and remained significantly higher than the first-trimester levels in the third trimester, whereas vitamin D3 increased by approximately 24.9% from the first to the second trimester. Vitamin E increased significantly by approximately 37.7% from the first to the second trimester and by a further 12.0% from the second to the third trimester, corresponding to an overall increase of 54.1%. Vitamin K increased significantly by approximately 66.7% from the second to the third trimester, with the third-trimester levels approximately 159.6% higher than first-trimester levels. Detailed results for maternal serum concentrations of fat-soluble vitamins during pregnancy are presented in Fig 3 and Table 2.

**Fig 3:**
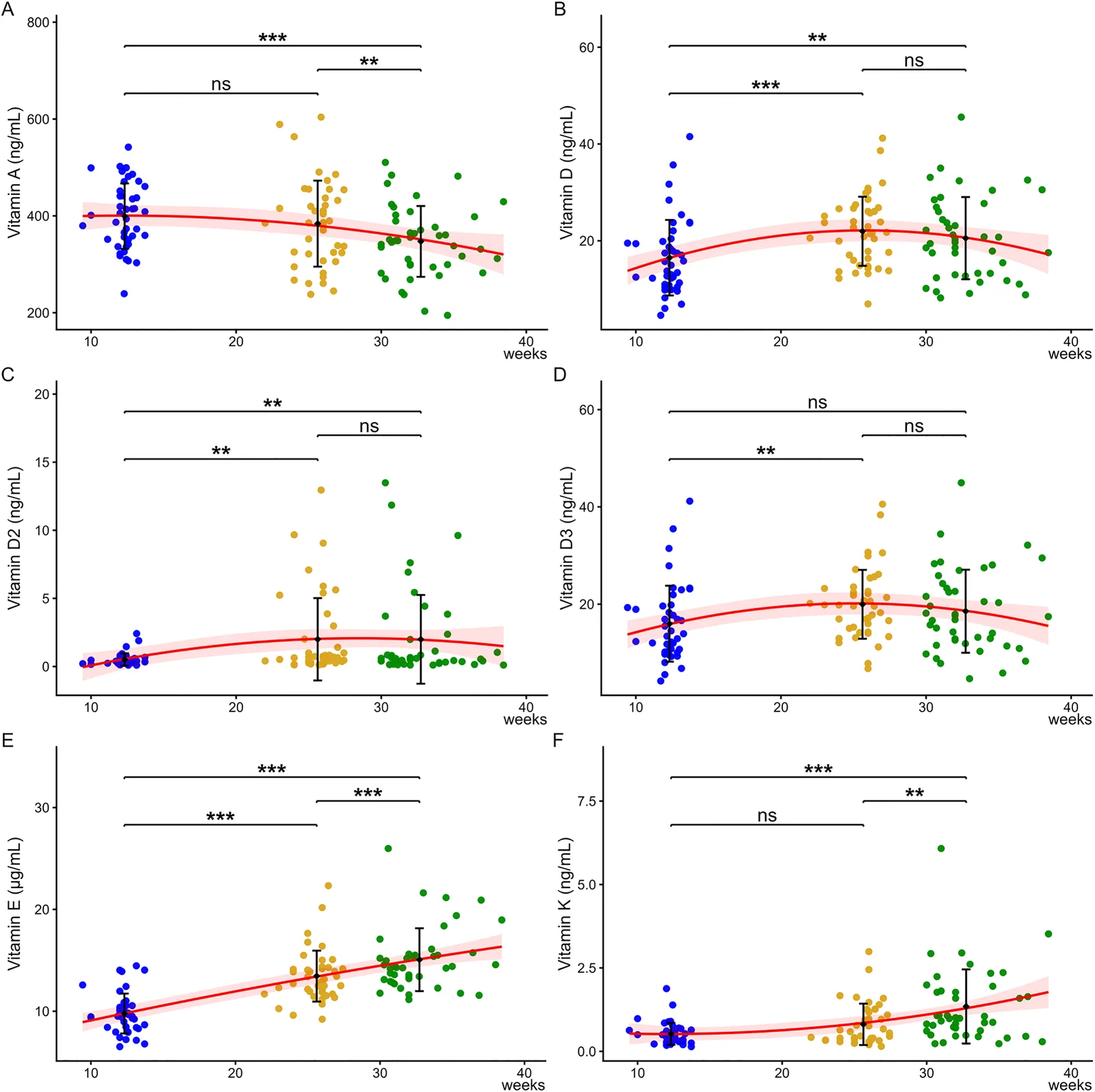
Serum fat-soluble vitamins during pregnancy. (A) Vitamin A; (B) Vitamin D; (C) Vitamin D2; (D) Vitamin D3; (E) Vitamin E; (F) Vitamin K. First trimester: blue; Second trimester: yellow; Third trimester: green. After Bonferroni correction, *P < 0.05, **P < 0.01, ***P < 0.001, and not significant (ns).

### 3.3. Fluctuations in water-soluble vitamins during pregnancy

Maternal B-vitamin levels exhibited distinct patterns of change throughout pregnancy. Vitamin B2 and B9 decreased significantly from the first to the second trimester, by approximately 21.3% and 30.7%, respectively. Third-trimester levels remained significantly lower than first-trimester levels, with overall reductions of 29.1% and 36.8%, respectively. Specific maternal serum concentrations of water-soluble vitamins during pregnancy are shown in Fig 4 and Table 2.

**Fig 4:**
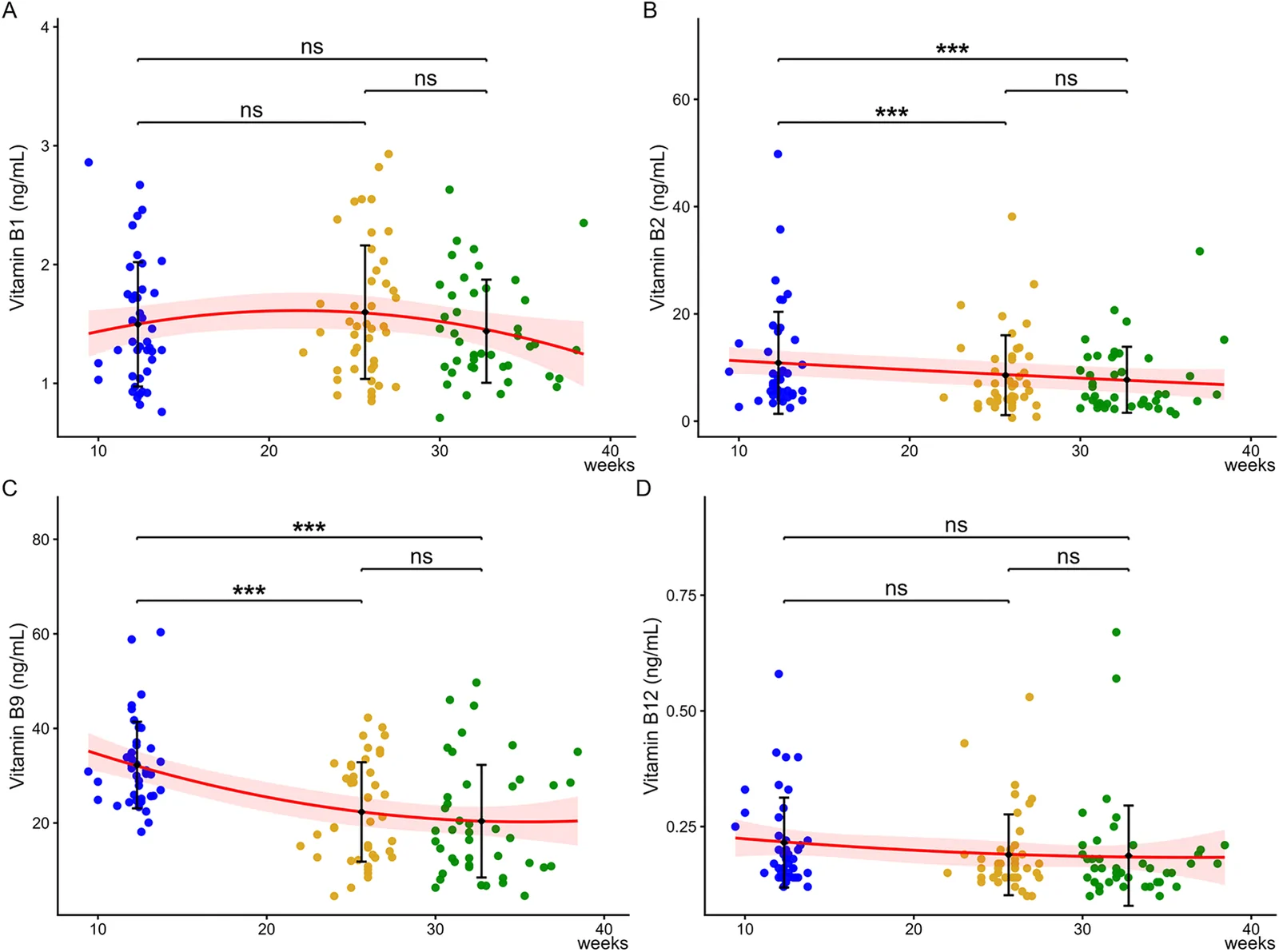
Serum water-soluble vitamins during pregnancy. (A) Vitamin B1; (B) Vitamin B2; (C) Vitamin B9; (D) Vitamin B12. First trimester: blue; Second trimester: yellow; Third trimester: green. After Bonferroni correction, *P < 0.05, **P < 0.01, ***P < 0.001, and not significant (ns).

### 3.4. Fluctuations in toxic element during pregnancy

Maternal whole-blood lead levels increased significantly by approximately 16.7% from the second to the third trimester. Trimester-specific changes and absolute concentrations of maternal whole-blood lead during pregnancy are presented in Fig 5 and Table 2.

**Fig 5:**
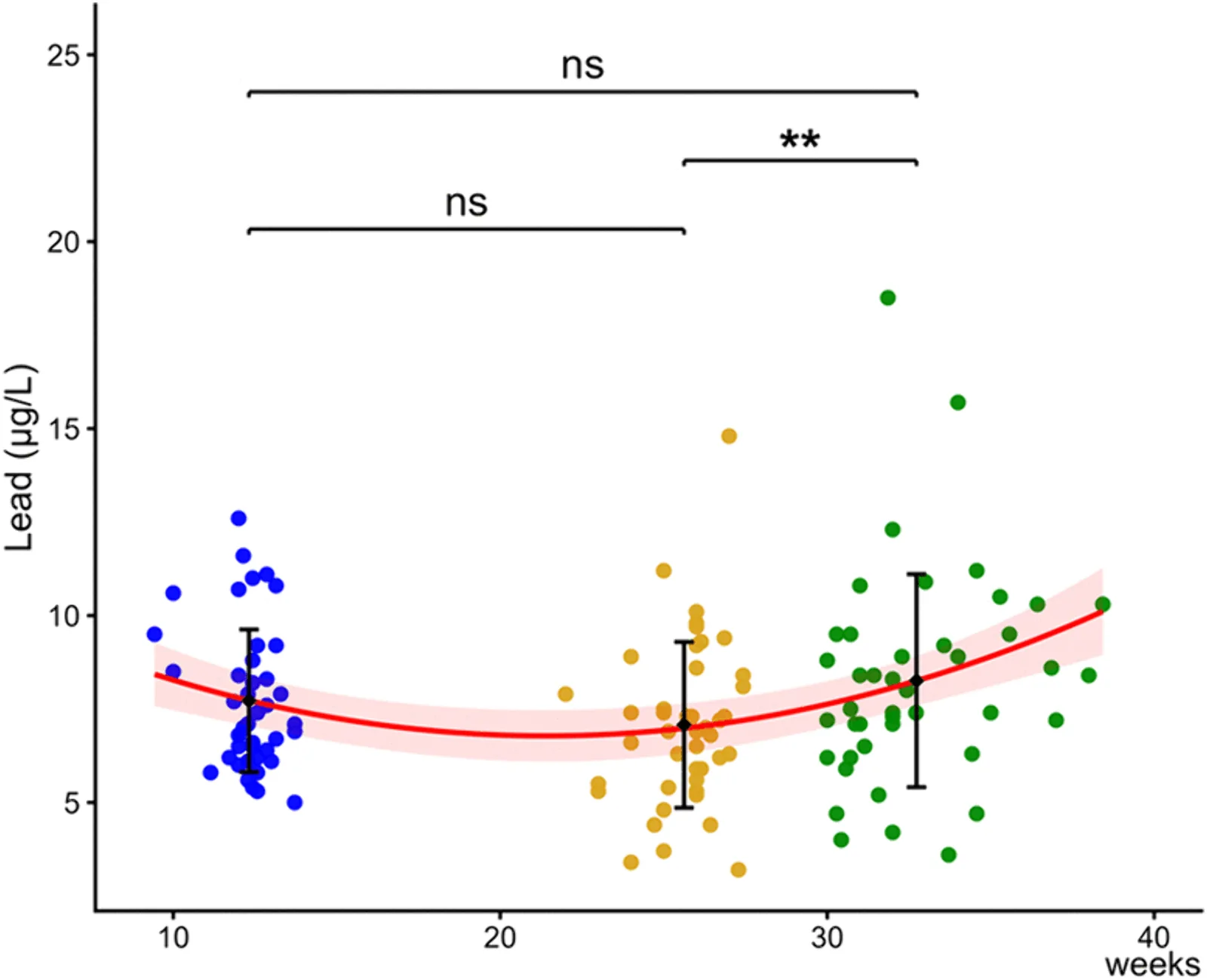
Toxic element lead during pregnancy. First trimester: blue; Second trimester: yellow; Third trimester: green. After Bonferroni correction, *P < 0.05, **P < 0.01, and not significant (ns).

## 4. Discussion

This prospective longitudinal cohort study in Lianyungang, an eastern coastal city in China, used repeated measurements across the first, second, and third trimesters to characterize gestational changes in 7 trace elements, 4 fat-soluble vitamins, and 4 B vitamins in healthy pregnant women. Overall, maternal circulating copper, manganese, vitamin E, and vitamin K increased with advancing gestation, while iron, magnesium, and zinc exhibited a broadly U-shaped pattern, with the lowest levels observed in the second trimester. Among the B vitamins, folate (vitamin B9) exhibited the most pronounced decline. By distinguishing overall gestational trajectories from trimester-specific changes, this repeated-measures design provides a more comprehensive depiction of gestational micronutrient fluctuations during healthy pregnancy than cross-sectional observations alone.

The transition from the first to the second trimester represented the period during which maternal micronutrient concentrations changed most markedly. During this interval, circulating copper increased, whereas iron, magnesium, zinc, and folate decreased. Previous studies from China [8], Spain [9] and Iranian [10] likewise reported progressive increases in maternal circulating copper during pregnancy, consistent with our findings. In contrast, the decline in iron, magnesium, and zinc during mid-pregnancy is biologically plausible and aligns with the physiological adaptations of pregnancy, including plasma volume expansion, increasing fetal nutrient demand, enhanced placental transfer, and maternal metabolic adjustment. The reduction in iron-related markers is also consistent with previous evidence showing substantially increased maternal iron requirements during gestation [11–13]. Earlier studies similarly documented decreases in magnesium and zinc during pregnancy, although the timing of the nadir and the magnitude of change varied across populations [8,14,15]. Such between-study differences likely reflect variation in ethnicity, dietary structure, environmental exposure, socioeconomic background, supplementation practices, and analytical methods [4,6,7]. Despite routine nutritional supplementation during pregnancy, serum folate concentrations declined progressively with advancing gestation. In addition to physiological factors such as gestational plasma volume expansion, placental transfer, and increased fetal developmental demand, reduced adherence to supplementation during pregnancy may also have contributed to the decline in folate levels observed in this cohort. Previous studies on maternal dietary patterns and longitudinal changes in folate-related metabolism support this interpretation [16].

Maternal micronutrient changes during the transition from the second to the third trimester showed a different pattern. Iron, magnesium, zinc and lead increased to some extent, whereas manganese, vitamin E, and vitamin K continued to rise. These findings indicate that gestational changes in circulating micronutrients are not monotonic and therefore should not be interpreted using a single reference interval across the entire pregnancy. The progressive increase in manganese observed in this study is broadly consistent with reports from Quebec, Beijing, and Taiwan, although absolute concentrations differed across populations [17–19]. Vitamin E also increased continuously, in line with previous longitudinal observations in normal pregnancy [20]. Because longitudinal data on vitamin K in healthy pregnancy remain limited, our results provide additional evidence for its gestational profile.

In this study, blood lead concentrations in all participants were below the CDC reference value of 3.5 μg/dL throughout gestation, indicating a low-level exposure profile. No participant reached the 5 μg/dL threshold for follow-up testing and assessment of potential exposure sources. Maternal blood lead showed a decline in the second trimester followed by a rebound in the third trimester. A possible explanation is that hemodilution during the second trimester may contribute to lower circulating lead concentrations, whereas increased maternal bone turnover during the third trimester could promote the release of previously stored lead from bone into the maternal circulation [21]. This pattern is clinically relevant because it suggests that endogenous lead exposure may increase with advancing gestation even in otherwise healthy pregnant women. Studies from Sweden and Poland reported blood lead levels broadly comparable to those observed in our cohort, whereas substantially higher levels were reported in French Guiana and Nigeria [22–25]. Maternal blood lead is therefore influenced not only by current environmental exposure but also by cumulative body burden from past exposure [5,21], underscoring the importance of minimizing lead exposure before conception whenever possible.

Taken together, these findings indicate that maternal micronutrient measurements should be interpreted in relation to gestational stage. The use of a single reference interval across the entire pregnancy may obscure normal physiological variation and lead to overestimation or underestimation of deficiency risk at different stages. In this cohort, maternal blood concentrations of copper, manganese, vitamin E, and vitamin K increased progressively throughout pregnancy, whereas maternal blood concentrations of iron, magnesium, and zinc reached their lowest levels in the second trimester. These longitudinal data, derived from local healthy pregnant women, may provide an important basis for future efforts to establish pregnancy-specific reference intervals and deficiency thresholds, particularly for trace elements such as copper and zinc [8].

This study has several limitations. First, circulating concentrations of many nutrients do not fully reflect total body stores. For example, circulating calcium, zinc, iron, and magnesium cannot accurately represent whole-body reserves. Previous studies have shown that peripheral blood contains only approximately 1% of total body calcium [26], 0.1% of zinc [27], 60%−70% of iron [28], and 0.8% of magnesium [29]. Second, strict inclusion criteria resulted in a notably small sample size of 43 healthy pregnant women without adverse pregnancy outcomes. Moreover, as this study focused on healthy women with uncomplicated pregnancies and normal neonatal outcomes, the findings may not be fully generalizable to other pregnant populations. Third, supplement use and dietary intake were not fully accounted for in the longitudinal analyses. Folic acid and calcium supplement use was self-reported, information on vitamin D supplementation was not systematically collected, and detailed dietary intake was not assessed. Thus, the observed changes, particularly in folate, calcium, and vitamin D, cannot be fully disentangled from the potential effects of diet and supplementation and should not be attributed solely to physiological changes during pregnancy. Nevertheless, peripheral blood remains the most feasible matrix for clinical monitoring, and longitudinal changes in circulating concentrations may still provide useful descriptive information for pregnancy-stage-specific interpretation.

## 5. Conclusion

Maternal micronutrient concentrations vary throughout gestation and should be interpreted according to gestational stage rather than against a single reference interval for the entire pregnancy. The pronounced variation in several analytes, particularly copper and zinc, highlights the importance of accounting for gestational age in nutritional assessment and laboratory interpretation. These findings should be confirmed in larger and more diverse populations before trimester- and region-specific reference intervals are established.

## Supporting information

S1 Table Analytical quality control data. (DOCX)

S2 Table Unadjusted and Bonferroni-adjusted pairwise P values for trimester-specific micronutrient comparisons. (DOCX)

S3 Table De-identified raw test data. (XLSX)
