# Supplementary material for: Fluctuations of micronutrients during pregnancy in Lianyungang, Jiangsu, China: A prospective longitudinal cohort study
Source: PLoS One. 2026 Oct 16;21(10):e0360813. doi: 10.1371/journal.pone.0360813 (PMC13674776; doi:10.1371/journal.pone.0360813)
Supplement: Supplementary file 1 [file pone.0360813.s001.docx]

| **Analyte** | **LOD** | **LOQ** | **Intra-assay CV (%)** | **Inter-assay CV (%)** |
| --- | --- | --- | --- | --- |
| Copper (μg/L) | 0.6837 | 1.5344 | 1.63-1.66 | 2.23-2.97 |
| Iron (mg/L) | 0.2830 | 0.6464 | 1.74-1.83 | 2.18-2.59 |
| Zinc (mg/L) | 0.0050 | 0.0057 | 1.65-1.71 | 2.20-2.64 |
| Calcium (mg/L) | 0.0166 | 0.0387 | 1.81-2.71 | 2.65-3.69 |
| Magnesium (mg/L) | 0.0009 | 0.0020 | 1.61-1.78 | 2.44-2.83 |
| Manganese (μg/L) | 0.0397 | 0.1038 | 1.97-2.02 | 3.69-3.95 |
| Lead (μg/L) | 0.0249 | 0.0675 | 1.66-1.75 | 2.31-2.52 |
| Vitamin A (ng/mL) | Not reported | 95 | 1.872-2.114 | 7.619-7.935 |
| Vitamin B1 (ng/mL) | Not reported | 0.93 | 4.84-5.31 | 6.24-7.35 |
| Vitamin B2 (ng/mL) | Not reported | 0.72 | 2.95-4.78 | 3.59-4.63 |
| Vitamin B9 (ng/mL) | Not reported | 1.00¹ | 8.80-9.97 | 4.65-5.50 |
| Vitamin B12 (ng/mL) | Not reported | 0.025² | Not reported | Not reported |
| Vitamin D2 (ng/mL) | Not reported | 1.50 | 3.60-10.42 | 4.99-9.02 |
| Vitamin D3 (ng/mL) | Not reported | 1.50 | 4.92-5.30 | 5.62-6.56 |
| Vitamin E (μg/mL) | Not reported | 0.762 | 3.323-4.383 | 5.753-6.794 |
| Vitamin K (ng/mL) | Not reported | 0.10 | 4.44-6.28 | 4.72-7.23 |

**S1 Table.** Analytical quality control data

**Note:** LOD, limit of detection; LOQ, limit of quantification.

¹ The value of 1.00 ng/mL represents the lower limit of the validated linear range.

² The value of 0.025 ng/mL represents the lower limit of the validated linear range.

Total vitamin D was calculated as the sum of vitamin D2 and vitamin D3.
