# Supplementary material for: Fluctuations of micronutrients during pregnancy in Lianyungang, Jiangsu, China: A prospective longitudinal cohort study
Source: PLoS One. 2026 Oct 16;21(10):e0360813. doi: 10.1371/journal.pone.0360813 (PMC13674776; doi:10.1371/journal.pone.0360813)
Supplement: Supplementary file 2 [file pone.0360813.s002.docx]

| **Micronutrient** | **P1** | | **P2** | | **P3** | |
| --- | --- | --- | --- | --- | --- | --- |
|  | **Unadj.** | **Bonferroni adj.** | **Unadj.** | **Bonferroni adj.** | **Unadj.** | **Bonferroni adj.** |
| Copper(μg/L) | <0.0001 | <0.0001 | <0.0001 | <0.0001 | 0.7516 | 1.0000 |
| Iron (mg/L) | <0.0001 | <0.0001 | 0.0381 | 0.1142 | 0.0006 | 0.0019 |
| Zinc (mg/L) | 0.0206 | 0.0618 | 0.0004 | 0.0013 | <0.0001 | <0.0001 |
| Calcium(mg/L) | 0.0146 | 0.0438 | 0.8211 | 1.0000 | 0.0260 | 0.0780 |
| Magnesium(mg/L) | <0.0001 | <0.0001 | <0.0001 | <0.0001 | 0.0019 | 0.0058 |
| Manganese(μg/L) | 0.0012 | 0.0036 | <0.0001 | <0.0001 | <0.0001 | <0.0001 |
| Lead (μg/L) | 0.0667 | 0.2002 | 0.1252 | 0.3755 | 0.0010 | 0.0030 |
| Vitamin A(ng/mL) | 0.2020 | 0.6059 | <0.0001 | 0.0001 | 0.0030 | 0.0091 |
| Vitamin B1(ng/mL) | 0.2130 | 0.6391 | 0.4769 | 1.0000 | 0.0521 | 0.1564 |
| Vitamin B2(ng/mL) | 0.0001 | 0.0004 | <0.0001 | <0.0001 | 0.1499 | 0.4496 |
| Vitamin B9(ng/mL) | <0.0001 | <0.0001 | <0.0001 | <0.0001 | 0.1726 | 0.5177 |
| Vitamin B12(ng/mL) | 0.1610 | 0.4829 | 0.1339 | 0.4016 | 0.9212 | 1.0000 |
| Vitamin D(ng/mL) | <0.0001 | <0.0001 | 0.0009 | 0.0027 | 0.2293 | 0.6879 |
| Vitamin D2(ng/mL) | 0.0013 | 0.0038 | 0.0013 | 0.0039 | 0.9938 | 1.0000 |
| Vitamin D3(ng/mL) | 0.0006 | 0.0018 | 0.0248 | 0.0743 | 0.2079 | 0.6237 |
| Vitamin E(μg/mL) | <0.0001 | <0.0001 | <0.0001 | <0.0001 | <0.0001 | <0.0001 |
| Vitamin K(ng/mL) | 0.0650 | 0.1951 | <0.0001 | <0.0001 | 0.0007 | 0.0022 |
| **Notes:** P1, second versus first trimester; P2, third versus first trimester; P3, third versus second trimester. | | | | | | |
|  | | | | | | |
|  | | | | | | |

**S2 Table.** Unadjusted and Bonferroni-adjusted pairwise P values for trimester-specific micronutrient comparisons
