# Supplementary material for: Fluctuations of micronutrients during pregnancy in Lianyungang, Jiangsu, China: A prospective longitudinal cohort study
Source: PLoS One. 2026 Oct 16;21(10):e0360813. doi: 10.1371/journal.pone.0360813 (PMC13674776; doi:10.1371/journal.pone.0360813)
Supplement: Supplementary file 3 [file pone.0360813.s005.docx]

**Response to the Academic Editor**
*1. Please avoid referring to lead (Pb) as a micronutrient. Pb is better discussed separately as a toxic metal or environmental exposure marker, and should not be grouped with essential trace elements and vitamins when discussing micronutrient reference ranges or deficiency thresholds.*

**Response:** Thank you for this important comment. We agree that Pb is not an essential micronutrient. We have therefore classified it separately as a toxic metal and environmental exposure marker and revised the relevant text, table (**Table 2**), figure title (**Fig 5**), and section headings accordingly (**Line 250-263**)**.**

*2. The final analysis was limited to women with uncomplicated pregnancies and normal neonatal findings at 42 days. This should be made clearer in the limitations, as it may affect how widely the findings can be applied to other pregnant populations.*

**Response:** Thank you for this comment. This study was specifically designed to characterize trimester-specific changes in trace elements and vitamins among healthy women with uncomplicated pregnancies and normal neonatal outcomes. Pregnant women with medical or obstetric complications were outside the predefined scope of the study. We have clarified this point in the **Limitation section** and emphasized that the findings are primarily applicable to healthy, low-risk pregnant populations. Future studies may investigate whether these patterns differ in women with complicated pregnancies **(Line 280-281)**.

*3. Several trace elements and vitamins were examined across the three trimesters. Please clarify how multiple comparisons were handled. If the adjustment was only applied to pairwise trimester comparisons, this should be stated clearly, and the results should be interpreted with some caution.*

**Response:** Thank you for this important comment. Bonferroni adjustment was applied to the three pairwise trimester comparisons for each trace element and vitamin. We have clarified this procedure in the Statistical Analysis section and reported both unadjusted and Bonferroni-adjusted P values in **S2 Table**. The Results and Discussion sections have also been revised to provide a more cautious interpretation of the findings.

*4. Please check that all figures are clear, complete, and submitted at adequate resolution. All panels should be fully visible and easy to read.*

**Response:** Thank you for this comment. We have carefully checked all figures to ensure that they are complete, clearly labeled, fully visible, and easy to read.

*5. Table 1 is included, but it would be useful to provide more information about the study population. If available, please add maternal and neonatal characteristics such as pre-pregnancy BMI, gestational weight gain, education or socioeconomic indicators, and neonatal birth weight. Please also check whether “Apgar score (10 min)” is correct, or whether the usual 1- and 5-minute Apgar scores should be reported.*
**Response:** Thank you for this helpful suggestion. We have expanded **Table 1** to include additional available maternal and neonatal characteristics, including pre-pregnancy BMI, gestational weight gain, education and socioeconomic indicators, and neonatal birth weight. We also rechecked the Apgar score variable. The reported value refers specifically to the Apgar score assessed at 10 minutes, as recorded in the clinical data, and is therefore not a typographical error.


**Response to the Reviewer**

*1. OVERVIEW
This prospective longitudinal cohort study characterizes trimester-specific changes in trace elements (Cu, Fe, Zn, Ca, Mg, Mn, Pb) and vitamins (A, B1, B2, B9, B12, D, E, K) in 43 healthy pregnant women from Lianyungang, China. The topic is clinically relevant and the longitudinal design is a genuine strength over previously reported cross-sectional studies. However, several methodological, analytical, and reporting issues must be addressed before the manuscript is suitable for publication. The concerns are detailed below and must be resolved in a revised submission.*

*2. MAJOR CONCERNS
2.1 Sample Size and Statistical Power
Issue: The final analysis included only 43 participants, representing a very small sample for establishing reference intervals or deficiency thresholds, which the authors claim as a primary contribution.
Required action: The authors must provide a formal a priori power calculation or sample size justification for the linear mixed-effects model analysis. If power is inadequate, the language around establishing reference intervals must be substantially toned down. Specifically, phrases such as "provide a basis for the future development of region-specific and trimester-specific reference intervals" must be explicitly qualified as preliminary and hypothesis-generating. The current sample size should also be clearly acknowledged as a major limitation in the Abstract, not only in the Discussion.*

**Response:** We thank the reviewer for this important comment. We agree that the final sample of 43 participants is insufficient to establish definitive trimester-specific reference intervals or deficiency thresholds. We have therefore revised the manuscript to more clearly characterize the study as an **exploratory longitudinal investigation** and have removed or qualified statements that could be interpreted as establishing reference intervals or diagnostic thresholds.

No formal a priori power calculation was performed at study initiation, because the sample size was primarily determined by study feasibility and the application of strict eligibility criteria. To address the reviewer’s concern and provide a quantitative sample-size justification for the longitudinal analysis, we subsequently performed sample-size calculations using **GLIMMPSE Version 3** under a three-time-point longitudinal repeated-measures framework (https://glimmpse.samplesizeshop.org/). A target power of **80%** and a type I error rate of **α = 0.05** were specified, and the **Hotelling-Lawley trace** was used to evaluate the overall trimester effect.

The present study evaluated **17 analytes, including trace elements and vitamins**. However, suitable published longitudinal data for formal sample-size estimation were available only for a subset of these analytes. Therefore, trimester-specific means and standard deviations for copper, zinc, iron, calcium, and magnesium were obtained from the longitudinal study by Tabrizi and Pakdel (2014) (Tabrizi FM, Pakdel FG. Serum Level of Some Minerals during Three Trimesters of Pregnancy in Iranian Women and Their Newborns: A Longitudinal Study. Indian J Clin Biochem. 2014 Apr;29(2):174-80. doi: 10.1007/s12291-013-0336-x. Epub 2013 May 21. PMID: 24757299; PMCID: PMC3990789), in which serum mineral concentrations were measured in the same cohort of 162 pregnant women across all three trimesters. Because that study did not report within-participant correlations among trimester-specific measurements, a relatively conservative within-subject correlation of **0.30** was assumed for the primary sample-size assessment.

The resulting sample-size estimates were as follows:

|  | Target power | Type I error α | Assumed within-subject correlation | N |
| --- | --- | --- | --- | --- |
| Cooper | 0.80 | 0.05 | 0.30 | 9 |
| Zinc | 0.80 | 0.05 | 0.30 | 18 |
| Iron | 0.80 | 0.05 | 0.30 | 27 |
| Calcium | 0.80 | 0.05 | 0.30 | 308 |
| Magnesium | 0.80 | 0.05 | 0.30 | 2068 |

For copper, zinc, and iron, which showed significant trimester-related changes in the reference study, the estimated required sample sizes were **9, 18, and 27 participants**, respectively. Thus, the final sample of 43 participants exceeded the largest estimated sample size among these analytes and provides reasonable statistical power to detect longitudinal changes of a magnitude comparable to those previously reported for copper, zinc, and iron.

In contrast, substantially larger sample sizes were estimated for calcium (**n = 308**) and magnesium (**n = 2068**). These large estimates reflect the very small trimester-related changes reported for these analytes, which were also statistically non-significant in the reference study. Therefore, although the present sample provides reasonable power for moderate-to-large longitudinal changes, we acknowledge that it may be underpowered to detect small changes in relatively stable micronutrients.

Importantly, these calculations do **not** indicate that the current sample size is sufficient to establish definitive reference intervals or deficiency thresholds. We have therefore revised the Abstract, Discussion, and Conclusion to explicitly state that the present findings should be regarded as **preliminary, exploratory, and hypothesis-generating**. Statements concerning reference intervals have been revised to indicate that the present data may inform the design of future adequately powered, large-scale studies aimed at establishing region- and gestational-stage-specific reference intervals, rather than establishing such intervals directly.

The limited sample size has also been explicitly identified as a major limitation in the **Abstract** and **Discussion** (**Lines 41-43 and 278-279**).

*2.2 Missing Numerical Data in the Results
Issue: The manuscript reports results almost entirely through figures (Figures 2, 3, 4) without providing any actual numerical data (means, standard deviations, confidence intervals, or p-values) in the text or in supplementary tables. This is a critical deficiency. PLOS ONE requires that data underlying figures be accessible and interpretable without relying solely on graphical estimation.
Required action: A comprehensive summary table (or supplementary data table) must be added reporting mean ± SD for each analyte in each trimester, the estimated marginal means from the linear mixed-effects models with 95% CIs, and Bonferroni-adjusted pairwise p-values for all significant comparisons. This is essential for reproducibility and for readers attempting to compare findings with other populations.*
**Response:** Thank you for highlighting this important issue. We have added a comprehensive **Table 2** reporting the mean ± SD and 95% confidence interval for each analyte in each trimester. In addition, **S2 Table** provides the Bonferroni-adjusted P values for all pairwise trimester comparisons. These additions provide the numerical data underlying **Figs 2-5** and improve the interpretability, reproducibility, and comparability of our findings.

*2.3 Table 1 is Missing from the Manuscript
Issue: The manuscript references "Table 1" (Anthropometric Characteristics of participants) but this table is not present anywhere in the submitted manuscript file. This is a critical omission.
Required action: Table 1 must be included in the revised manuscript. It should report: maternal age, pre-pregnancy BMI, gestational weight gain, parity, educational level, and neonatal birth weight/Apgar scores, as these are important for contextualizing the findings and assessing the generalizability of the cohort.*
**Response:** Thank you for identifying this omission. **Table 1** has now been included in the revised manuscript and expanded to report the relevant maternal and neonatal characteristics, including maternal age, pre-pregnancy BMI, gestational weight gain, parity, educational level, neonatal birth weight, and Apgar score (**Line 448**).

*2.4 Incomplete Reporting of Vitamin D Subtypes
Issue: The study reports vitamin D, D2, and D3 as separate analytes (Figures 3B, 3C, 3D). However, total vitamin D (25-hydroxyvitamin D) is the sum of D2 and D3 and should be explained as such. It is biologically unusual to report D2 and D3 as independent fractions without discussing their dietary and metabolic origins in this cohort, especially given the folic acid and calcium supplementation protocol used.
Required action: Clarify in the Methods whether total vitamin D was measured as the sum of D2 and D3 or independently. Discuss the observed D2 elevation in the second trimester in the context of dietary sources or supplementation. State whether any participants took vitamin D supplements (this appears to have been excluded but needs explicit confirmation).*

**Response:** Thank you for this important comment. We have clarified in the **Method** that total vitamin D was calculated as the sum of vitamin D₂ and vitamin D₃ rather than measured independently. All participants followed a normal diet in accordance with dietary guidelines, and no vitamin D supplements were prescribed as part of the study protocol. Because detailed dietary intake data were not collected, the specific dietary or metabolic basis for the increase in vitamin D₂ during the second trimester could not be determined (**Line 96-97 and 99-100**).

*2.5 Lead (Pb) Reporting Requires Expanded Contextualisation
Issue: The inclusion of blood lead as an analyte is noted, but the clinical and public health implications are inadequately discussed. The authors mention that lead levels rebounded in the third trimester due to possible bone turnover yet do not compare their observed lead values against internationally recognized safety thresholds (e.g., CDC reference value of 3.5 μg/dL, or WHO guidance). The study population's mean and range for blood lead are not reported numerically in the text.
Required action: Report mean ± SD blood lead values by trimester numerically. Compare observed values with current international safety thresholds. If all values are below safety thresholds, this should be stated explicitly to contextualize the public health relevance. If any values exceeded thresholds, this must be disclosed and discussed. Add a sentence clarifying whether the lead findings have any immediate clinical implications for this specific cohort.*

**Response:** Thank you for this important comment. We have added the trimester-specific mean ± SD blood lead concentrations to Table 2 and expanded the Discussion to contextualize these findings using international reference and action levels. All individual blood lead concentrations in this cohort were below the CDC blood lead reference value of 3.5 μg/dL and the WHO action threshold of 5 μg/dL. These results indicate a generally low lead burden among the healthy, low-risk pregnant women included in this study. Although blood lead concentrations increased in the third trimester, potentially reflecting enhanced maternal bone turnover, they remained below these reference and action levels. Therefore, the observed changes do not indicate an immediate need for clinical intervention in this cohort. Nevertheless, because no level of lead exposure is considered entirely risk-free, continued efforts to minimize maternal lead exposure remain warranted (**Line 250-253,437**).

*2.6 Confounders Not Accounted For in Analysis
Issue: The authors acknowledge in the Discussion (lines 247-248) that "dietary intake, supplement adherence, environmental exposure, and socioeconomic factors were not included in the analysis" but they do not explain why, nor do they discuss how this omission might bias the reported trajectories. Given that folic acid and calcium supplementation were recommended to all participants (lines 84-85), the observed folate decline and calcium pattern may partly reflect variable supplementation adherence, which cannot be disentangled with the current analytical approach.
Required action: The limitations section must be expanded to explicitly acknowledge that the reported micronutrient trajectories represent total circulating levels and cannot distinguish physiological change from supplement-related change. The authors should discuss how adherence variability might specifically affect the folate, calcium, and vitamin D findings. Consider whether any sensitivity analysis (e.g., excluding participants with evidence of non-adherence) was possible.*

**Response:** Thank you for this important comment. This study was originally designed to characterize trimester-specific changes in circulating trace elements and vitamins among healthy women with uncomplicated pregnancies. Standardized dietary data, detailed supplement records, and environmental exposures were not collected prospectively and could not be reliably reconstructed retrospectively. Socioeconomic characteristics are presented in **Table 1** (**Line 448**).

We have therefore clarified in the **Limitation** that the observed trajectories reflect total circulating concentrations and cannot distinguish physiological changes from effects related to diet or supplementation. Variability in adherence to folic acid and calcium supplementation may have influenced the folate and calcium patterns, while unrecorded dietary intake or self-initiated supplementation may also have affected vitamin D levels.

A sensitivity analysis based on supplement adherence was considered but was not feasible because objective adherence data, such as pill counts or medication records, were unavailable. This limitation has now been stated explicitly (**Line 282-283**).

Despite the absence of quantitative dietary data, we believe that our findings provide valuable real-world descriptive evidence regarding the trajectories of these micronutrients during pregnancy under routine clinical care and standard supplementation recommendations. Nevertheless, future large-scale studies incorporating detailed dietary assessments and objective adherence monitoring are needed to disentangle the complex contributions of physiological changes, dietary intake, and supplementation to maternal micronutrient status.

*2.7 Discrepancy in Participant Numbers
Issue: The Figure 1 flowchart shows 130 women screened, 51 eligible, and 45 with blood samples collected, while the text (lines 105-108) states 43 completed all assessments. The flowchart shows N=45 at blood preservation, then N=44 at parturition, then N=43 at neonatal follow-up. However, the Methods text states 51 were enrolled and 43 completed - the intermediate steps between 51 and 43 are not described in the text (only in the figure). This creates a reporting inconsistency.
Required action: Reconcile the flowchart and the text. The Methods section must explicitly describe each exclusion step (e.g., n=2 excluded at parturition for which reasons; n=1 excluded at 42-day follow-up for which reason). The number enrolled (51) vs. the number eligible in Figure 1 (51) vs. the 130 screened should also be explained more clearly in the Methods text.*
**Response:** Thank you for identifying this inconsistency. We have reconciled the participant numbers in **Fig 1** and the **Method** section. The revised Methods now clearly describe the full recruitment process, including the 130 women screened, the 51 eligible and enrolled participants, and each subsequent exclusion or loss to follow-up leading to the final analytic cohort of 43 women. The reasons for exclusion due to pregnancy complications, at delivery, and during the 42-day neonatal follow-up have also been specified. Figure 1 and the corresponding text are now fully consistent (**Line 82-87,113-117**).

*3. MINOR CONCERNS
1. The Abstract states that the cohort was established at "6-8 weeks of gestation" (line 29), but the Methods specify blood sampling began at "12.3 ± 0.9 weeks" in the first trimester (line 113). The enrollment and the first blood draw are clearly at different gestational ages, but the Abstract implies they coincide. Clarify this distinction in the Abstract.*
**Response:** Thank you for highlighting this ambiguity. We have revised the Abstract to clearly distinguish the timing of participant enrollment from that of the first blood collection. The cohort was established at 6-8 weeks of gestation, whereas the first blood sample was collected at 12.3 ± 0.9 weeks during the first trimester. The corresponding statement in the **Abstract** has been revised accordingly (**Line 34-35**).

*2. Line 84 states that "folic acid (0.4-0.8 mg/day) and calcium (600 mg/day) were recommended." PLOS ONE requires that dietary supplement protocols be described precisely. Were all 43 participants confirmed to have received these supplements? Was adherence monitored or self-reported? Was any other supplementation possible (e.g., prenatal multivitamins)? These details must be specified.*
**Response:** Thank you for this comment. All 43 participants confirmed that they took folic acid (0.4-0.8 mg/day) and calcium (600 mg/day) as recommended. Adherence was assessed by self-report, and participants reported following the supplementation recommendations and maintaining a normal diet in accordance with dietary guidelines. However, adherence was not objectively monitored using pill counts or medication diaries. These details have now been clarified in the **Method** section (**Line 89-92,99-100**).

*3. The statistical software version is reported as "R version 4.5.3" (line 140). As of the submission date this version does not appear to have been released. Please verify and correct the R version number.*
**Response:** Thank you for identifying this error. We carefully rechecked the statistical software used in the analysis and confirmed that the correct version was R version 4.5.0. The incorrect version number has been corrected in the revised manuscript (**Line 149**).

*4. Lines 143-146 describe outlier handling: one manganese value (42.8 μg/L) was excluded. The rationale for this specific threshold should be stated more clearly. Was this based on a Grubbs test, interquartile range rule, or clinical judgment alone? The text should also state how many potential outliers were identified in total and how many were retained versus excluded.*
**Response:** Thank you for this important comment. We have clarified the method used for outlier identification in the revised manuscript. Potential outliers were assessed separately for each trimester using the boxplot interquartile range (IQR) rule, with values below (Q1-1.5/times IQR) or above (Q3+1.5/times IQR) considered outliers. Only one potential outlier was identified across all measurements: a first-trimester manganese concentration of 42.8 μg/L. This value exceeded the corresponding upper cutoff and was therefore excluded from the statistical analysis. No other potential outliers were identified; thus, one value was excluded and none were retained as outliers (**Line 152-155**).

*5. The manuscript refers to "ICP-MS" and "UHPLC-MS/MS" without specifying whether method validation data (e.g., limits of detection, limits of quantification, intra- and inter-assay CVs) are available. While the authors state that "quality control materials were included in each analytical batch" (line 135), PLOS ONE encourages transparency in analytical performance. A supplementary table with method validation parameters would strengthen the manuscript.*
**Response:** Thank you for this constructive suggestion. To improve transparency regarding analytical performance, we have added the method validation parameters for ICP-MS and UHPLC-MS/MS to **S1 Table.** The table now includes the limits of detection, limits of quantification, and intra- and inter-assay coefficients of variation for all measured trace elements and vitamins. Quality control materials included in each analytical batch are also described in the **Method** section.

*6. The Discussion (lines 193-194) states findings are "consistent" with studies from China, Spain, and Finland for copper, citing references [8-10]. However, reference [10] (Kiilholma et al., 1984) is over 40 years old and was conducted in a small-for-gestational-age pregnancy cohort, making it a questionable comparator for a healthy pregnancy cohort. The relevance of this reference should be reconsidered or justified.*
**Response:** Thank you for this important comment. We agree that the previous reference was not an appropriate comparator for our healthy pregnancy cohort. We have therefore replaced reference [10] with a 2013 study of maternal copper levels among Iranian pregnant women, which provides a more relevant comparison with our study population. (**Line 223**).

*7. Lines 243-244 cite reference [25] to support the claim that peripheral blood contains "approximately 1% of total body calcium." However, reference [25] (Powell et al., 1978) is cited in the manuscript's reference list as a study on serum ferritin and iron stores in haemochromatosis, which appears to be an incorrect citation for the calcium statement. Please verify and correct the reference.*
**Response:** Thank you for raising this concern. We carefully rechecked the citation and the corresponding reference list. Reference [25] in our manuscript is Beto’s article, The Role of Calcium in Human Aging, rather than the study by Powell et al. on serum ferritin and iron stores. Beto reported that approximately 99% of total body calcium is found in the bones and teeth, whereas approximately 1% is present in serum. Therefore, Reference [25] was intended to support the statement regarding calcium distribution. In the revised manuscript, this reference has been renumbered as Reference [26]. To avoid any potential confusion, we have carefully checked the citation numbering and ensured that the correct reference is cited in both the text and the reference list (**Line 277**).

*8. In Figure 2 legend (lines 390-393) and throughout the Results section, statistical significance is denoted as *P<0.05, **P<0.01, ***P<0.001. However, these are described as post-Bonferroni corrected values. The uncorrected p-values and the exact adjusted p-values for key comparisons should ideally be reported in a supplementary table for transparency.*
**Response:** Thank you for this helpful suggestion. For greater transparency, **S2 Table** provides both the unadjusted *P* values and the exact Bonferroni-adjusted *P* values for the relevant pairwise comparisons.

*9. The keywords list uses inconsistent punctuation: some terms are separated by semicolons and others by commas ("Pregnancy; Micronutrients, Trace elements; Vitamins; Longitudinal Cohort, Maternal blood"). Please standardize keyword formatting to follow PLOS ONE style (semicolons throughout).*
**Response:** Thank you for noting this inconsistency. The keywords have been revised to use semicolons throughout, in accordance with PLOS ONE style.

*10. The manuscript does not mention whether gestational age was confirmed by ultrasound at each trimester blood draw or only at enrollment. Given the stated gestational week ranges for blood collection (lines 113-117), it is important to confirm the method of gestational age assignment at each time point.*
**Response:** Thank you for this comment. Gestational age was confirmed by B-ultrasound only at enrollment (6-8 weeks of gestation), based on the presence of a fetal bud and fetal cardiac activity. The gestational ages at subsequent blood collection visits were determined according to the gestational age established at enrollment and were not reconfirmed by ultrasound at each visit (**line 122**).

*4. LANGUAGE AND PRESENTATION
The manuscript is generally readable but would benefit from careful English language editing before resubmission. The following specific issues were noted:
• Line 48: "Pregnancy is a unique physiological state, characterized by plasma volume expansion..." - the comma after "state" is unnecessary and disrupts the sentence.
• Line 56: "D-dimer levels rise progressively throughout pregnancy[1-3]" - the citation placement appears to span references that are not all D-dimer studies; verify citation accuracy.
• Lines 160-162: The Results section uses very brief sentences without quantitative data. Each result statement should be supported by at least the direction and approximate magnitude of change, even if full tables appear as supplementary material.
• The Conclusion section (lines 253-259) is essentially a restatement of the Discussion's final paragraph. Consider condensing the Conclusion to 3-4 sentences that focus only on the most novel and actionable findings.*
**Response:** Thank you for these helpful comments. We have carefully revised the manuscript accordingly. The unnecessary comma in Line 48 has been removed, and the references supporting the statement on D-dimer levels have been corrected and verified. The Results section has been expanded to include the direction and approximate magnitude of the observed changes, together with the relevant quantitative data. In addition, the Conclusion has been condensed to focus on the principal novel findings and their clinical and research implications.

5. RECOMMENDATION
This manuscript addresses a clinically relevant question using an appropriate longitudinal design. With substantial revision addressing the concerns above- particularly the addition of numerical result tables, inclusion of Table 1, reconciliation of the participant flowchart, and toning down of claims about establishing reference intervals -this work could make a useful contribution to the literature on pregnancy-specific micronutrient physiology. I recommend Major Revision.
