# Supplementary material for: Fluctuations of micronutrients during pregnancy in Lianyungang, Jiangsu, China: A prospective longitudinal cohort study
Source: PLoS One. 2026 Oct 16;21(10):e0360813. doi: 10.1371/journal.pone.0360813 (PMC13674776; doi:10.1371/journal.pone.0360813)
Supplement: Supplementary file 4 [file pone.0360813.s006.docx]

**Point-by-point Response to Reviewer 1 and the Editor’s Comments**

**Comments partially addressed**

1. *Table 2 now provides mean (SD) and 95% CIs, and S2 includes the pairwise P values. However, it is not clear whether the values in Table 2 are descriptive means or estimated marginal means from the mixed-effects models, as requested by Reviewer 1. Please clarify this and provide the model-derived estimates if applicable.*

**Response:** Thank you for pointing this out. We have added estimated marginal means (EMMs) with 95% confidence intervals from the mixed-effects models to **Table 2**, while retaining the descriptive means (SDs). Pairwise comparisons and corresponding P values from the mixed-effects models are provided in S2 Table.

2. *In the response letter, the authors state that all 43 participants took the recommended folic acid and calcium supplements and that adherence was assessed by self-report. The Methods, however, still state only that these supplements were “recommended.” Please revise the Methods to reflect the information provided in the response.*

**Response:** Thank you for pointing this out. We have revised the Participants subsection of the Methods as follows: “After enrollment, participants were advised to take folic acid (0.4-0.8 mg/day) and calcium (600 mg/day). Adherence to supplementation was assessed by self-report during follow-up, and all 43 participants included in the final analysis reported taking both supplements as recommended. Information on self-initiated use of vitamin D or other nutritional supplements was not systematically collected.”

3. *The response letter provides the IQR criterion used for outlier identification and states that only one value was excluded. This information is not fully described in the Methods. Please revise the relevant section so that the procedure and number of excluded observations are clear.*

**Response:** Thank you for this comment. We have revised the Statistical Analysis subsection to clearly describe the outlier identification procedure and the number of excluded observations. The revised text reads: “Potential outliers were identified using Tukey’s 1.5 × IQR rule (values below Q1 - 1.5 × IQR or above Q3 + 1.5 × IQR). Only one manganese concentration (42.8 μg/L) was excluded and treated as missing in the manganese analysis, while other observations were retained.”

**Comments not fully incorporated into the manuscript**

1. *The response letter explains that the observed micronutrient changes cannot be clearly separated from possible effects of diet and supplementation, particularly for folate, calcium, and vitamin D. This point is only briefly mentioned in the manuscript. Please expand the limitations section accordingly.*

**Response:** Thank you for this important comment. We have revised the original text as follows: “Third, supplement use and dietary intake were not fully accounted for in the longitudinal analyses. Folic acid and calcium supplement use was self-reported, information on vitamin D supplementation was not systematically collected, and detailed dietary intake was not assessed. Thus, the observed changes, particularly in folate, calcium, and vitamin D, cannot be fully disentangled from the potential effects of diet and supplementation and should not be attributed solely to physiological changes during pregnancy.”

2. *Please clarify the statements regarding vitamin D and other supplement use. The response states that no vitamin D supplements were prescribed, while the Methods state that “no other nutritional supplements were used,” and another response refers to possible self-initiated supplementation. These statements should be made consistent.*

**Response:** Thank you for pointing out this inconsistency. We agree that our previous wording was not sufficiently precise. Folic acid and calcium were recommended as part of routine antenatal care, and all 43 participants included in the final analysis reported taking these supplements. However, information on self-initiated use of vitamin D or other nutritional supplements was not systematically collected or verified. Therefore, we cannot exclude the possibility that some participants may have used such supplements independently. We have revised the Methods and corresponding statements throughout the manuscript to clarify this point and have removed the statement that “no other nutritional supplements were used.”

3. *Please recheck the references related to mineral distribution. The statement that approximately 0.1% of total body zinc is present in peripheral blood cites Reference 27, but this reference concerns iron stores. Please correct the citation and verify the relevant reference numbering.*

**Response:** Thank you for pointing this out. We have corrected the citation for the statement regarding zinc distribution. The original reference has now been replaced with the article “Zinc Homeostasis in Humans” by King et al (DOI: 10.1093/jn/130.5.1360S). We have also carefully rechecked all references throughout the manuscript to ensure the accuracy of the citations.
