# Supplementary material for: Fluctuations of micronutrients during pregnancy in Lianyungang, Jiangsu, China: A prospective longitudinal cohort study
Source: PLoS One. 2026 Oct 16;21(10):e0360813. doi: 10.1371/journal.pone.0360813 (PMC13674776; doi:10.1371/journal.pone.0360813)
Supplement: Peer review history

# Peer review: Decision Letter 0

**DOI:** 10.1371/journal.pone.0360813.r001

**Authors:** Iman Al-Saleh

29 Jun 2026

PLOS One

Dear Dr. Zhao,

Thank you for submitting your manuscript to PLOS ONE. After careful consideration, we feel that it has merit but does not fully meet PLOS ONE’s publication criteria as it currently stands. Therefore, we invite you to submit a revised version of the manuscript that addresses the points raised during the review process.

====================================

Thank you for submitting your manuscript to the journal.

Based on the detailed feedback provided by the external reviewer and my own editorial assessment of the manuscript, I am inviting you to submit a **major revision** .

The manuscript addresses a relevant topic and has strengths, particularly the longitudinal design. However, several important methodological, analytical, and reporting issues need to be addressed before the manuscript can be considered further.

Please revise the manuscript carefully in response to all reviewer and editorial comments provided below. In your response letter, please provide a detailed point-by-point reply to each comment and indicate where changes have been made in the revised manuscript. Please note that the revised manuscript may be sent for further review if needed.

=================================

Please submit your revised manuscript by Aug 28 2026 11:59PM. If you will need more time than this to complete your revisions, please reply to this message or contact the journal office at plosone@plos.org. When you're ready to submit your revision, log on to https://www.editorialmanager.com/pone/ and select the 'Submissions Needing Revision' folder to locate your manuscript file.

Please include the following items when submitting your revised manuscript:

- A letter that responds to each point raised by the academic editor and reviewer(s). You should upload this letter as a separate file labeled 'Response to Reviewers'.
- A marked-up copy of your manuscript that highlights changes made to the original version. You should upload this as a separate file labeled 'Revised Manuscript with Track Changes'.
- An unmarked version of your revised paper without tracked changes. You should upload this as a separate file labeled 'Manuscript'.

If you would like to make changes to your financial disclosure, please include your updated statement in your cover letter. Guidelines for resubmitting your figure files are available below the reviewer comments at the end of this letter.

If applicable, we recommend that you deposit your laboratory protocols in protocols.io to enhance the reproducibility of your results. Protocols.io assigns your protocol its own identifier (DOI) so that it can be cited independently in the future. For instructions see: https://journals.plos.org/plosone/s/submission-guidelines#loc-laboratory-protocols. Additionally, PLOS ONE offers an option for publishing peer-reviewed Lab Protocol articles, which describe protocols hosted on protocols.io. Read more information on sharing protocols at https://plos.org/protocols?utm_medium=editorial-email&utm_source=authorletters&utm_campaign=protocols.

As the corresponding author, your ORCID iD is verified in the submission system and will appear in the published article. PLOS supports the use of ORCID, and we encourage all coauthors to register for an ORCID iD and use it as well. Please encourage your coauthors to verify their ORCID iD within the submission system before final acceptance, as unverified ORCID iDs will not appear in the published article. *Only*  the individual author can complete the verification step; PLOS staff *cannot*  verify ORCID iDs on behalf of authors.

We look forward to receiving your revised manuscript.

Kind regards,

Iman Al-Saleh

Academic Editor

PLOS One

Journal Requirements:

When submitting your revision, we need you to address these additional requirements.

1. Please ensure that your manuscript meets PLOS ONE's style requirements, including those for file naming. The PLOS ONE style templates can be found at

https://journals.plos.org/plosone/s/file?id=wjVg/PLOSOne_formatting_sample_main_body.pdf and

https://journals.plos.org/plosone/s/file?id=ba62/PLOSOne_formatting_sample_title_authors_affiliations.pdf.

2. Please include your tables as part of your main manuscript and remove the individual files. Please note that supplementary tables (should remain/ be uploaded) as separate "supporting information" files.

3. Thank you for stating in your Funding Statement:

“This study was supported by the Science Foundation of Kangda College of Nanjing Medical University (grant no. KD2024KYJJ073), Key Research Project on Hospital Management Innovation (grant no. JSYGY-2-2024-81) and Key Project on Innovative Research in Health Management (grant no. K202406).”

Please provide an amended statement that declares *all* the funding or sources of support (whether external or internal to your organization) received during this study, as detailed online in our guide for authors at http://journals.plos.org/plosone/s/submit-now.  Please also include the statement “There was no additional external funding received for this study.” in your updated Funding Statement.

Please include your amended Funding Statement within your cover letter. We will change the online submission form on your behalf.

4. Thank you for stating the following financial disclosure:

“This study was supported by the Science Foundation of Kangda College of Nanjing Medical University (grant no. KD2024KYJJ073), Key Research Project on Hospital Management Innovation (grant no. JSYGY-2-2024-81) and Key Project on Innovative Research in Health Management (grant no. K202406).”

Please state what role the funders took in the study.  If the funders had no role, please state: "The funders had no role in study design, data collection and analysis, decision to publish, or preparation of the manuscript."

If this statement is not correct you must amend it as needed.

Please include this amended Role of Funder statement in your cover letter; we will change the online submission form on your behalf.

5. In the online submission form you indicate that your data is not available for proprietary reasons and have provided a contact point for accessing this data. Please note that your current contact point is a co-author on this manuscript. According to our Data Policy, the contact point must not be an author on the manuscript and must be an institutional contact, ideally not an individual. Please revise your data statement to a non-author institutional point of contact, such as a data access or ethics committee, and send this to us via return email. Please also include contact information for the third party organization, and please include the full citation of where the data can be found.

6. PLOS requires an ORCID iD for the corresponding author in Editorial Manager on papers submitted after December 6th, 2016. Please ensure that you have an ORCID iD and that it is validated in Editorial Manager. To do this, go to ‘Update my Information’ (in the upper left-hand corner of the main menu), and click on the Fetch/Validate link next to the ORCID field. This will take you to the ORCID site and allow you to create a new iD or authenticate a pre-existing iD in Editorial Manager.

7. Please be informed that funding information should not appear in the Acknowledgments section or other areas of your manuscript. We will only publish funding information present in the Funding Statement section of the online submission form. Please remove any funding-related text from the manuscript.

8. If the reviewer comments include a recommendation to cite specific previously published works, please review and evaluate these publications to determine whether they are relevant and should be cited. There is no requirement to cite these works unless the editor has indicated otherwise.

**Editor Comments** :

In addition to the reviewer’s comments, I would like the authors to consider the following points when revising the manuscript:

1. Please avoid referring to lead (Pb) as a micronutrient. Pb is better discussed separately as a toxic metal or environmental exposure marker, and should not be grouped with essential trace elements and vitamins when discussing micronutrient reference ranges or deficiency thresholds.

2. The final analysis was limited to women with uncomplicated pregnancies and normal neonatal findings at 42 days. This should be made clearer in the limitations, as it may affect how widely the findings can be applied to other pregnant populations.

3. Several trace elements and vitamins were examined across the three trimesters. Please clarify how multiple comparisons were handled. If the adjustment was only applied to pairwise trimester comparisons, this should be stated clearly, and the results should be interpreted with some caution.

4. Please check that all figures are clear, complete, and submitted at adequate resolution. All panels should be fully visible and easy to read.

5. Table 1 is included, but it would be useful to provide more information about the study population. If available, please add maternal and neonatal characteristics such as pre-pregnancy BMI, gestational weight gain, education or socioeconomic indicators, and neonatal birth weight. Please also check whether “Apgar score (10 min)” is correct, or whether the usual 1- and 5-minute Apgar scores should be reported.

[Note: HTML markup is below. Please do not edit.]

Reviewers' comments:

Reviewer's Responses to Questions

**Comments to the Author**

1. Is the manuscript technically sound, and do the data support the conclusions?

Reviewer #1: Partly

**********

2. Has the statistical analysis been performed appropriately and rigorously?

Reviewer #1: No

**********

3. Have the authors made all data underlying the findings in their manuscript fully available?

The PLOS Data policy

Reviewer #1: No

**********

4. Is the manuscript presented in an intelligible fashion and written in standard English?

Reviewer #1: Yes

**********

**Reviewer #1** : 1. OVERVIEW

This prospective longitudinal cohort study characterizes trimester-specific changes in trace elements (Cu, Fe, Zn, Ca, Mg, Mn, Pb) and vitamins (A, B1, B2, B9, B12, D, E, K) in 43 healthy pregnant women from Lianyungang, China. The topic is clinically relevant and the longitudinal design is a genuine strength over previously reported cross-sectional studies. However, several methodological, analytical, and reporting issues must be addressed before the manuscript is suitable for publication. The concerns are detailed below and must be resolved in a revised submission.

2. MAJOR CONCERNS

2.1 Sample Size and Statistical Power

Issue: The final analysis included only 43 participants, representing a very small sample for establishing reference intervals or deficiency thresholds, which the authors claim as a primary contribution.

Required action: The authors must provide a formal a priori power calculation or sample size justification for the linear mixed-effects model analysis. If power is inadequate, the language around establishing reference intervals must be substantially toned down. Specifically, phrases such as "provide a basis for the future development of region-specific and trimester-specific reference intervals" must be explicitly qualified as preliminary and hypothesis-generating. The current sample size should also be clearly acknowledged as a major limitation in the Abstract, not only in the Discussion.

2.2 Missing Numerical Data in the Results

Issue: The manuscript reports results almost entirely through figures (Figures 2, 3, 4) without providing any actual numerical data (means, standard deviations, confidence intervals, or p-values) in the text or in supplementary tables. This is a critical deficiency. PLOS ONE requires that data underlying figures be accessible and interpretable without relying solely on graphical estimation.

Required action: A comprehensive summary table (or supplementary data table) must be added reporting mean ± SD for each analyte in each trimester, the estimated marginal means from the linear mixed-effects models with 95% CIs, and Bonferroni-adjusted pairwise p-values for all significant comparisons. This is essential for reproducibility and for readers attempting to compare findings with other populations.

2.3 Table 1 is Missing from the Manuscript

Issue: The manuscript references "Table 1" (Anthropometric Characteristics of participants) but this table is not present anywhere in the submitted manuscript file. This is a critical omission.

Required action: Table 1 must be included in the revised manuscript. It should report: maternal age, pre-pregnancy BMI, gestational weight gain, parity, educational level, and neonatal birth weight/Apgar scores, as these are important for contextualizing the findings and assessing the generalizability of the cohort.

2.4 Incomplete Reporting of Vitamin D Subtypes

Issue: The study reports vitamin D, D2, and D3 as separate analytes (Figures 3B, 3C, 3D). However, total vitamin D (25-hydroxyvitamin D) is the sum of D2 and D3 and should be explained as such. It is biologically unusual to report D2 and D3 as independent fractions without discussing their dietary and metabolic origins in this cohort, especially given the folic acid and calcium supplementation protocol used.

Required action: Clarify in the Methods whether total vitamin D was measured as the sum of D2 and D3 or independently. Discuss the observed D2 elevation in the second trimester in the context of dietary sources or supplementation. State whether any participants took vitamin D supplements (this appears to have been excluded but needs explicit confirmation).

2.5 Lead (Pb) Reporting Requires Expanded Contextualisation

Issue: The inclusion of blood lead as an analyte is noted, but the clinical and public health implications are inadequately discussed. The authors mention that lead levels rebounded in the third trimester due to possible bone turnover yet do not compare their observed lead values against internationally recognized safety thresholds (e.g., CDC reference value of 3.5 μg/dL, or WHO guidance). The study population's mean and range for blood lead are not reported numerically in the text.

Required action: Report mean ± SD blood lead values by trimester numerically. Compare observed values with current international safety thresholds. If all values are below safety thresholds, this should be stated explicitly to contextualize the public health relevance. If any values exceeded thresholds, this must be disclosed and discussed. Add a sentence clarifying whether the lead findings have any immediate clinical implications for this specific cohort.

2.6 Confounders Not Accounted For in Analysis

Issue: The authors acknowledge in the Discussion (lines 247-248) that "dietary intake, supplement adherence, environmental exposure, and socioeconomic factors were not included in the analysis" but they do not explain why, nor do they discuss how this omission might bias the reported trajectories. Given that folic acid and calcium supplementation were recommended to all participants (lines 84-85), the observed folate decline and calcium pattern may partly reflect variable supplementation adherence, which cannot be disentangled with the current analytical approach.

Required action: The limitations section must be expanded to explicitly acknowledge that the reported micronutrient trajectories represent total circulating levels and cannot distinguish physiological change from supplement-related change. The authors should discuss how adherence variability might specifically affect the folate, calcium, and vitamin D findings. Consider whether any sensitivity analysis (e.g., excluding participants with evidence of non-adherence) was possible.

2.7 Discrepancy in Participant Numbers

Issue: The Figure 1 flowchart shows 130 women screened, 51 eligible, and 45 with blood samples collected, while the text (lines 105-108) states 43 completed all assessments. The flowchart shows N=45 at blood preservation, then N=44 at parturition, then N=43 at neonatal follow-up. However, the Methods text states 51 were enrolled and 43 completed — the intermediate steps between 51 and 43 are not described in the text (only in the figure). This creates a reporting inconsistency.

Required action: Reconcile the flowchart and the text. The Methods section must explicitly describe each exclusion step (e.g., n=2 excluded at parturition for which reasons; n=1 excluded at 42-day follow-up for which reason). The number enrolled (51) vs. the number eligible in Figure 1 (51) vs. the 130 screened should also be explained more clearly in the Methods text.

3. MINOR CONCERNS

1. The Abstract states that the cohort was established at "6-8 weeks of gestation" (line 29), but the Methods specify blood sampling began at "12.3 ± 0.9 weeks" in the first trimester (line 113). The enrollment and the first blood draw are clearly at different gestational ages, but the Abstract implies they coincide. Clarify this distinction in the Abstract.

2. Line 84 states that "folic acid (0.4-0.8 mg/day) and calcium (600 mg/day) were recommended." PLOS ONE requires that dietary supplement protocols be described precisely. Were all 43 participants confirmed to have received these supplements? Was adherence monitored or self-reported? Was any other supplementation possible (e.g., prenatal multivitamins)? These details must be specified.

3. The statistical software version is reported as "R version 4.5.3" (line 140). As of the submission date this version does not appear to have been released. Please verify and correct the R version number.

4. Lines 143-146 describe outlier handling: one manganese value (42.8 μg/L) was excluded. The rationale for this specific threshold should be stated more clearly. Was this based on a Grubbs test, interquartile range rule, or clinical judgment alone? The text should also state how many potential outliers were identified in total and how many were retained versus excluded.

5. The manuscript refers to "ICP-MS" and "UHPLC-MS/MS" without specifying whether method validation data (e.g., limits of detection, limits of quantification, intra- and inter-assay CVs) are available. While the authors state that "quality control materials were included in each analytical batch" (line 135), PLOS ONE encourages transparency in analytical performance. A supplementary table with method validation parameters would strengthen the manuscript.

6. The Discussion (lines 193-194) states findings are "consistent" with studies from China, Spain, and Finland for copper, citing references [8-10]. However, reference [10] (Kiilholma et al., 1984) is over 40 years old and was conducted in a small-for-gestational-age pregnancy cohort, making it a questionable comparator for a healthy pregnancy cohort. The relevance of this reference should be reconsidered or justified.

7. Lines 243-244 cite reference [25] to support the claim that peripheral blood contains "approximately 1% of total body calcium." However, reference [25] (Powell et al., 1978) is cited in the manuscript's reference list as a study on serum ferritin and iron stores in haemochromatosis, which appears to be an incorrect citation for the calcium statement. Please verify and correct the reference.

8. In Figure 2 legend (lines 390-393) and throughout the Results section, statistical significance is denoted as *P<0.05, **P<0.01, ***P<0.001. However, these are described as post-Bonferroni corrected values. The uncorrected p-values and the exact adjusted p-values for key comparisons should ideally be reported in a supplementary table for transparency.

9. The keywords list uses inconsistent punctuation: some terms are separated by semicolons and others by commas ("Pregnancy; Micronutrients, Trace elements; Vitamins; Longitudinal Cohort, Maternal blood"). Please standardize keyword formatting to follow PLOS ONE style (semicolons throughout).

10. The manuscript does not mention whether gestational age was confirmed by ultrasound at each trimester blood draw or only at enrollment. Given the stated gestational week ranges for blood collection (lines 113-117), it is important to confirm the method of gestational age assignment at each time point.

4. LANGUAGE AND PRESENTATION

The manuscript is generally readable but would benefit from careful English language editing before resubmission. The following specific issues were noted:

• Line 48: "Pregnancy is a unique physiological state, characterized by plasma volume expansion..." — the comma after "state" is unnecessary and disrupts the sentence.

• Line 56: "D-dimer levels rise progressively throughout pregnancy[1-3]" — the citation placement appears to span references that are not all D-dimer studies; verify citation accuracy.

• Lines 160-162: The Results section uses very brief sentences without quantitative data. Each result statement should be supported by at least the direction and approximate magnitude of change, even if full tables appear as supplementary material.

• The Conclusion section (lines 253-259) is essentially a restatement of the Discussion's final paragraph. Consider condensing the Conclusion to 3-4 sentences that focus only on the most novel and actionable findings.

5. RECOMMENDATION

This manuscript addresses a clinically relevant question using an appropriate longitudinal design. With substantial revision addressing the concerns above — particularly the addition of numerical result tables, inclusion of Table 1, reconciliation of the participant flowchart, and toning down of claims about establishing reference intervals — this work could make a useful contribution to the literature on pregnancy-specific micronutrient physiology. I recommend Major Revision.

**********

what does this mean?). If published, this will include your full peer review and any attached files.

If you choose “no”, your identity will remain anonymous but your review may still be made public.

**Do you want your identity to be public for this peer review?**  For information about this choice, including consent withdrawal, please see our Privacy Policy

Reviewer #1: **Yes:**  Adebola Emmanuel Orimadegun

**********

[NOTE: If reviewer comments were submitted as an attachment file, they will be attached to this email and accessible via the submission site. Please log into your account, locate the manuscript record, and check for the action link "View Attachments". If this link does not appear, there are no attachment files.]

To ensure your figures meet our technical requirements, please review our figure guidelines: https://journals.plos.org/plosone/s/figures

You may also use PLOS’s free figure tool, NAAS, to help you prepare publication quality figures: https://journals.plos.org/plosone/s/figures#loc-tools-for-figure-preparation.

NAAS will assess whether your figures meet our technical requirements by comparing each figure against our figure specifications.

# Author comment: Author response to Decision Letter 1

**DOI:** 10.1371/journal.pone.0360813.r002

24 Aug 2026

Thank you for the opportunity to revise our manuscript. We have carefully addressed all comments raised by the Academic Editor and Reviewer and have revised the manuscript accordingly.

In response to the Academic Editor’s comments, Pb has been clearly distinguished from essential micronutrients and is now discussed separately as a toxic metal/environmental exposure marker. We have also clarified the generalizability of the study population, specified the use of Bonferroni correction for pairwise trimester comparisons, checked all figures for clarity and completeness, and expanded Table 1 to include additional maternal and neonatal characteristics.

In response to the Reviewer’s major comments, we clarified the exploratory nature of the study and provided a quantitative sample-size justification based on published longitudinal data and GLIMMPSE calculations. We also toned down statements regarding the establishment of reference intervals or deficiency thresholds and explicitly acknowledged the limited sample size. A comprehensive Table 2 and supplementary tables have been added to provide numerical data, confidence intervals, and both unadjusted and Bonferroni-adjusted P values.

We further clarified vitamin D measurements and supplementation, expanded the interpretation of blood lead concentrations, acknowledged the potential effects of diet and supplement adherence, reconciled all participant numbers between the Methods and flowchart, and revised the relevant methodological and reporting details.

All minor comments were also addressed, including clarification of enrollment versus blood-collection timing, supplementation adherence, correction of the R software version, clarification of outlier handling, addition of analytical validation parameters, correction and verification of references, reporting of exact adjusted P values, standardization of keywords, clarification of gestational-age assessment, and English-language editing.

In addition, as requested by the Editorial Office, the Funding Statement has been updated to include all sources of support: the Science Foundation of Kangda College of Nanjing Medical University (grant no. KD2024KYJJ073), the Key Research Project on Hospital Management Innovation (grant no. JSYGY-2-2024-81), and the Key Project on Innovative Research in Health Management (grant no. K202406). There was no additional external funding received for this study. The Role of Funders statement has also been added: “The funders had no role in study design, data collection and analysis, decision to publish, or preparation of the manuscript.” These statements have been included in the revised cover letter as requested.

A detailed point-by-point response to all comments has been provided in the Response to Reviewers document. We sincerely thank the Academic Editor and Reviewer for their constructive comments, which have helped us substantially improve the clarity, methodological transparency, and overall quality of the manuscript.

Attachment Submitted filename: Response to Reviewers.docx

# Editor report: Decision Letter 1

**DOI:** 10.1371/journal.pone.0360813.r003

**Authors:** Iman Al-Saleh

16 Sep 2026

Dear Dr. Zhao,

Thank you for submitting the revised manuscript. The manuscript has improved considerably, and most of the comments from Reviewer 1 and the editor have been addressed. A few points, however, still need clarification or have not yet been fully reflected in the revised manuscript.

**Comments partially addressed**

1. Table 2 now provides mean (SD) and 95% CIs, and S2 includes the pairwise P values. However, it is not clear whether the values in Table 2 are descriptive means or estimated marginal means from the mixed-effects models, as requested by Reviewer 1. Please clarify this and provide the model-derived estimates if applicable.
2. In the response letter, the authors state that all 43 participants took the recommended folic acid and calcium supplements and that adherence was assessed by self-report. The Methods, however, still state only that these supplements were “recommended.” Please revise the Methods to reflect the information provided in the response.
3. The response letter provides the IQR criterion used for outlier identification and states that only one value was excluded. This information is not fully described in the Methods. Please revise the relevant section so that the procedure and number of excluded observations are clear.

**Comments not fully incorporated into the manuscript**

1. The response letter explains that the observed micronutrient changes cannot be clearly separated from possible effects of diet and supplementation, particularly for folate, calcium, and vitamin D. This point is only briefly mentioned in the manuscript. Please expand the limitations section accordingly.
2. Please clarify the statements regarding vitamin D and other supplement use. The response states that no vitamin D supplements were prescribed, while the Methods state that “no other nutritional supplements were used,” and another response refers to possible self-initiated supplementation. These statements should be made consistent.
3. Please recheck the references related to mineral distribution. The statement that approximately 0.1% of total body zinc is present in peripheral blood cites Reference 27, but this reference concerns iron stores. Please correct the citation and verify the relevant reference numbering.

The remaining comments from Reviewer 1 and the editor appear to have been satisfactorily addressed. Please submit a revised manuscript together with a point-by-point response indicating where the above changes have been made.

Please submit your revised manuscript by Oct 16 2026 11:59PM. If you will need more time than this to complete your revisions, please reply to this message or contact the journal office at plosone@plos.org. When you're ready to submit your revision, log on to https://www.editorialmanager.com/pone/ and select the 'Submissions Needing Revision' folder to locate your manuscript file.

Please include the following items when submitting your revised manuscript:

- A letter that responds to each point raised by the academic editor and reviewer(s). You should upload this letter as a separate file labeled 'Response to Reviewers'.
- A marked-up copy of your manuscript that highlights changes made to the original version. You should upload this as a separate file labeled 'Revised Manuscript with Track Changes'.
- An unmarked version of your revised paper without tracked changes. You should upload this as a separate file labeled 'Manuscript'.

If you would like to make changes to your financial disclosure, please include your updated statement in your cover letter. Guidelines for resubmitting your figure files are available below the reviewer comments at the end of this letter.

If applicable, we recommend that you deposit your laboratory protocols in protocols.io to enhance the reproducibility of your results. Protocols.io assigns your protocol its own identifier (DOI) so that it can be cited independently in the future. For instructions see: https://journals.plos.org/plosone/s/submission-guidelines#loc-laboratory-protocols. Additionally, PLOS One offers an option for publishing peer-reviewed Lab Protocol articles, which describe protocols hosted on protocols.io. Read more information on sharing protocols at https://plos.org/protocols?utm_medium=editorial-email&utm_source=authorletters&utm_campaign=protocols.

As the corresponding author, your ORCID iD is verified in the submission system and will appear in the published article. PLOS supports the use of ORCID, and we encourage all coauthors to register for an ORCID iD and use it as well. Please encourage your coauthors to verify their ORCID iD within the submission system before final acceptance, as unverified ORCID iDs will not appear in the published article. *Only*  the individual author can complete the verification step; PLOS staff *cannot*  verify ORCID iDs on behalf of authors.

We look forward to receiving your revised manuscript.

Kind regards,

Iman Al-Saleh

Academic Editor

PLOS One

Journal Requirements:

1. If the reviewer comments include a recommendation to cite specific previously published works, please review and evaluate these publications to determine whether they are relevant and should be cited. There is no requirement to cite these works unless the editor has indicated otherwise.

2. Please review your reference list to ensure that it is complete and correct. If you have cited papers that have been retracted, please include the rationale for doing so in the manuscript text, or remove these references and replace them with relevant current references. Any changes to the reference list should be mentioned in the rebuttal letter that accompanies your revised manuscript. If you need to cite a retracted article, indicate the article’s retracted status in the References list and also include a citation and full reference for the retraction notice.

[Note: HTML markup is below. Please do not edit.]

[NOTE: If reviewer comments were submitted as an attachment file, they will be attached to this email and accessible via the submission site. Please log into your account, locate the manuscript record, and check for the action link "View Attachments". If this link does not appear, there are no attachment files.]

To ensure your figures meet our technical requirements, please review our figure guidelines: https://journals.plos.org/plosone/s/figures

You may also use PLOS’s free figure tool, NAAS, to help you prepare publication quality figures: https://journals.plos.org/plosone/s/figures#loc-tools-for-figure-preparation.

NAAS will assess whether your figures meet our technical requirements by comparing each figure against our figure specifications.

# Author comment: Author response to Decision Letter 2

**DOI:** 10.1371/journal.pone.0360813.r004

30 Sep 2026

Dear Editor,

Thank you for the opportunity to revise our manuscript (PONE-D-26-21186R1). We sincerely appreciate the Editor and Reviewer 1 for their careful evaluation and constructive comments.

In this revision, we have addressed all remaining concerns. The major changes include adding the model-derived estimated marginal means (EMMs) with 95% confidence intervals to Table 2, clarifying folic acid and calcium supplementation and outlier handling in the Methods, expanding the limitations regarding dietary intake and supplement use, and correcting the reference concerning zinc distribution. We have also carefully checked the manuscript to ensure consistency and accuracy throughout.

A detailed point-by-point response has been provided, and all corresponding changes have been incorporated into the revised manuscript.

We are grateful to the Editor and Reviewer for their helpful comments and appreciate the time and effort devoted to reviewing our manuscript.

Sincerely,

Cheng Zhao

on behalf of all authors

Attachment Submitted filename: Response_to_Reviewers_auresp_2.docx

# Editor report: Decision Letter 2

**DOI:** 10.1371/journal.pone.0360813.r005

**Authors:** Iman Al-Saleh

1 Oct 2026

Fluctuations of Micronutrients during Pregnancy in Lianyungang, Jiangsu, China: A Prospective Longitudinal Cohort Study

PONE-D-26-21186R2

Dear Dr. Zhao,

We’re pleased to inform you that your manuscript has been judged scientifically suitable for publication and will be formally accepted for publication once it meets all outstanding technical requirements.

Within one week, you’ll receive an e-mail detailing the required amendments. When these have been addressed, you’ll receive a formal acceptance letter and your manuscript will be scheduled for publication.

An invoice will be generated when your article is formally accepted. Please note, if your institution has a publishing partnership with PLOS and your article meets the relevant criteria, all or part of your publication costs will be covered. Please make sure your user information is up-to-date by logging into Editorial Manager at Editorial Manager® and clicking the ‘Update My Information' link at the top of the page. For questions related to billing, please contact billing support.

If your institution or institutions have a press office, please notify them about your upcoming paper to help maximize its impact. If they’ll be preparing press materials, please inform our press team as soon as possible -- no later than 48 hours after receiving the formal acceptance. Your manuscript will remain under strict press embargo until 2 pm Eastern Time on the date of publication. For more information, please contact onepress@plos.org.

Kind regards,

Iman Al-Saleh

Academic Editor

PLOS One

Additional Editor Comments (optional):

The authors have adequately addressed my previous comments. I have no further concerns and recommend acceptance.

Reviewers' comments:

# Editor report: Acceptance letter

**DOI:** 10.1371/journal.pone.0360813.r006

**Authors:** Iman Al-Saleh

PONE-D-26-21186R2

PLOS One

Dear Dr. Zhao,

I'm pleased to inform you that your manuscript has been deemed suitable for publication in PLOS One. Congratulations! Your manuscript is now being handed over to our production team.

At this stage, our production department will prepare your paper for publication. This includes ensuring the following:

* All references, tables, and figures are properly cited

* All relevant supporting information is included in the manuscript submission,

* There are no issues that prevent the paper from being properly typeset

You will receive further instructions from the production team, including instructions on how to review your proof when it is ready. Please keep in mind that we are working through a large volume of accepted articles, so please give us a few days to review your paper and let you know the next and final steps.

Lastly, if your institution or institutions have a press office, please let them know about your upcoming paper now to help maximize its impact. If they'll be preparing press materials, please inform our press team within the next 48 hours. Your manuscript will remain under strict press embargo until 2 pm Eastern Time on the date of publication. For more information, please contact onepress@plos.org.

You will receive an invoice from PLOS for your publication fee after your manuscript has reached the completed accept phase. If you receive an email requesting payment before acceptance or for any other service, this may be a phishing scheme. Learn how to identify phishing emails and protect your accounts at https://explore.plos.org/phishing.

If we can help with anything else, please email us at customercare@plos.org.

Thank you for submitting your work to PLOS One and supporting open access.

Kind regards,

PLOS One Editorial Office Staff

on behalf of

Dr. Iman Al-Saleh

Academic Editor

PLOS One
